# Deeply Saddening Life Events Play a Carcinogenic Role by Inducing Mutations in *ALOX12* and *FKBP5* Genes

**DOI:** 10.3390/genes15121531

**Published:** 2024-11-28

**Authors:** Ahmet Kutluhan, Osman Z. Topak, Hakan Akca, Elvan Tokgun, Osman I. Ozdel, Sevda Yilmaz, Ugur Sungurtekin, Ergun Erdem, Arzu Yaren

**Affiliations:** 1 Department of Otolaryngology, Head and Neck Surgery, Pamukkale University School of Medicine, Denizli 20070, Turkey; 2 Department of Psychiarty, Pamukkale University School of Medicine, Denizli 20070, Turkey; 3 Department of Medical Genetic, Pamukkale University School of Medicine, Denizli 20070, Turkey; 4 Department of Surgery, Pamukkale University School of Medicine, Denizli 20070, Turkey; 5 Department of Medical Oncology, Pamukkale University School of Medicine, Denizli 20070, Turkey

**Keywords:** psychological stress, Stressful Life Experiences, cancer, *ALOX12*, *FKBP5*, mutations

## Abstract

Background/Objectives: In the past few decades, many studies have been conducted to find out that psychological stress and cancer are closely linked. Moreover, it was reported that stress can induce mutations in gene level. Therefore, in this study we want to examine a relationship between stressful life events, gene mutation and cancer. Methods: Stressful Life Experiences Screening (SLES), Hospital Anxiety and Depression Scale (HADS) and the Coping with Stress Style Scale (CSS) were applied to the participants to examine relationship between stress and cancer. Results: NGS results showed higher level of mutations accumulated on *FKBP5* and *ALOX12* genes in cancer patients who were exposed to stressful life events. The expression status of *ALOX12* and *FKBP5* genes on patients with or without cancer and several cancer cell lines demonstrated that both *ALOX12* and *FKBP5* mRNA levels were downregulated only in cancer patients and cancer cell lines but not in cancer free control groups. Re-created overexpression of the WT-*ALOX12* and WT-*FKBP5* extremely inhibited cellular growth, cellular invasion in cancer cell lines, tumor growth in xenograft model too. Conclusions: Our results indicate that Stressful Life Experiences may induce cancer development by increased somatic mutations in *ALOX12* and *FKBP5* genes.

## 1. Introduction

Stress can be defined as a response to external stimulations that are associated with neuroendocrine changes. It is primarily regulated by two pathways: the sympathetic nervous system (SNS) and hypothalamic–pituitary–adrenal (HPA) axis [1]. Stress is caused by environmental or individual factors and has many physiological effects. Psychological stress describes what people experience when they are under mental, physical, or emotional pressure. Stressor factors can arise from people’s daily responsibilities and routines, including work, family, and finances. Other stressors include external factors such as early life adversity, exposure to certain environmental conditions, poverty, discrimination, and inequities in the social determinants of health. Chronic stress is a predisposing factor for insomnia, gastrointestinal disorders, anxiety, cardiovascular disease, and cancer [2]. Many studies have been conducted to determine the relationship between cancer and stress, and it is argued that the physiological effects of stress may play an important role in the development and progression of cancer.

Cancer is a major public health and economic problem of the 21st century. Approximately one in six deaths worldwide is caused by cancer (Global cancer statistics 2022: GLOBOCAN estimates of global incidence and mortality for 36 cancers in 185 countries). According to GLOBOCAN, there were nearly 20 million new cancer cases and 9.7 million cancer-related deaths in 2022. Therefore, it is essential to expand our knowledge about cancer [3].

Many environmental and genetic factors play important roles in the development of cancer. While factors such as cell type, stage at diagnosis, aggressiveness, and proximity to vital organs play a role in the progression of cancer, a person’s psychological state and stress factors are also thought to be important. The most important physiological effect of psychological stress in cancer is damage to the immune system. Stress causes the immune system to become dysfunctional, allowing the cancer to escape the immune system, facilitating angiogenesis and metastasis. In addition, psychological stress affects a person’s daily routine. An unbalanced diet, a sedentary lifestyle, and smoking and alcohol consumption, which can increase under stressful conditions, also contribute to the development of cancer. Chronic stress gives rise to an increase in the levels of stress hormones such as catecholamines and corticosteroids. Increased stress hormones lead to the development and progression of cancer via several mechanisms, for instance, by causing DNA damage, increasing *P53* degradation, and affecting the tumor microenvironment [2]. Several studies have revealed that chronic stress has an influence on tumorigenesis. Lin Q et al. showed that the activation of β2-adrenergic receptors (β2-AR), which are the main effectors of the SNS, accelerate tumor development by activating downstream ERK1/2 in colorectal cancer [4]. In another study, Zhou et al. revealed that elevated norepinephrine levels resulting from chronic stress activated β2-adrenergic receptors and enhanced VEGF/FGF2-mediated breast cancer angiogenesis [5]. It was shown that the PD-1 expression in tumor-infiltrating NK cells increased due to depression in hepatocellular carcinoma mice and affected their cytotoxicity negatively [6]. Numerous studies have been carried out like those above, and all of them indicated that stress causes tumor development and increases metastatic ability.

Importantly, stressful life events affect people in many ways. Some people experience no change, some develop post-traumatic stress disorder, and some exhibit subsyndromal symptoms or other changes in physical and mental health. Given this range of responses, event history is important [7]. Multiple studies indicate that older adults may be better at handling acute stress than their younger counterparts. Moreover, older people have a reduced ability to cope with stress [8,9]. Conversely, cancer incidence increases dramatically with age [10]. For example, cancer is diagnosed in fewer than 25 per 100,000 Americans under age 20, making up just 1 percent of all cancer diagnoses, according to the National Cancer Institute (NCI). That rate grows to 350 cases per 100,000 for Americans between the ages of 45 and 49. For adults aged 60 and older, that rate nearly triples to 1000 cases of cancer per 100,000 people [11]. Studies have shown relationships between cancer and stressful life events at various levels [12]. Another important aspect is the coping strategies of individuals with stress, which can take various forms. It has been reported that the coping styles of individuals in stressful situations or life events can intervene in some outcomes such as anxiety, depression, psychological distress, and somatic complaints [13,14]. Studies have shown that cancer survivors seem to use different coping strategies that vary throughout the survivorship process. Besides individual personalities, accepting the diagnosis and engaging in physical activities that provide social and emotional support seem to have a significant effect on coping among cancer survivors [15]. Understanding the benefits of coping strategies in severe stress conditions and during the survivorship course seems to be essential to planning modern care in cancer treatment. Chronic stress can cause physical changes in the body. While chronic stress has not yet been proven to increase the risk of developing cancer, it may affect the tumor’s ability to grow and spread. This is likely due, in part, to the release of norepinephrine. In a series of experiments, the tumors of mice exposed to stressful situations (such as isolation) were more likely to grow and spread. Researchers also found that breast cancer patients that reported using β blockers had a better chance of surviving treatment without relapse than those who did not [16].

FK506 binding protein 5 (*FKBP5*), which is also called *FKBP51*, is a 51 kDa FK506 binding protein that belongs to a family of immunophilins [17]. The activation of progesterone receptors, androgen receptors, and glucocorticoid receptors triggers *FKBP5* expression [18]. It was observed that the expression of *FKBP5* is both upregulated and downregulated in human cancers [19].

Arachidonate lipoxygenase12 (*ALOX12*) is a lipoxygenase-type enzyme that converts arachidonic acid to 12-hydroxyeicosatetraenoic acid (12-HETE) through the action of glutathione peroxide [20]. It has been reported that *ALOX12* and 12-HETE mediate cancer proliferation, survival, and metastasis [21,22]. The overexpression of *ALOX12* has been addressed in several tumor tissues, including prostrate and colorectal cancer [23,24].

The aim of the present study was to fill the depicted gaps and investigate the relationships between psychological stress and cancer. The aims of the study were (a) to explore and compare the possible relationship between coping styles for deeply saddening life events and cancer in cancer patients; (b) to evaluate traumatic events leading to increased gene mutations in cancer patients; and (c) to determine which gene/genes can play a role in psychological-stress-induced cancer progression.

The following innovations characterize our study: firstly, we directly assessed the relationship between coping styles for deeply saddening life events and cancer; secondly, we investigated the relationship with psychological stress gene mutations using next-generation sequencing; thirdly, this is the first study to indicate a relationship between psychological stress, *ALOX12* and *FKBP5* genes, and cancer progression.

## 2. Material and Methods

### 2.1. Participants

A total of 52 patients were enrolled in this study (Table 1). Our patient cohort included patients who had only recently learned that they had cancer, and had not received any medications before filling out any form for stress evaluation or providing their blood samples for the NGS study. A socio-demographic data form was filled in for each participant. The data of the patients consisted of sex, age, smoking history, stressful life events, anxiety, depression levels, and type of cancer. Stressful Life Experiences Screening (SLES), the Hospital Anxiety and Depression Scale (HADS), and the Styles of Coping with Stress Scale (CSS) were applied to the participants to examine the relationship between stress and cancer. A structured clinical interview form (SCID-5) for DSM [25] was also applied to the participants by a psychiatry specialist.

### 2.2. Measures

#### 2.2.1. Stressful Life Experiences Screening (SLES)

SLES is a 20-item scale which determines the degree of important events in a patient’s life history. It was designed to provide a fast and reliable measure of the history of potentially stressful experiences. It was developed by Stamm et al. [7,26]. The participants give answers between 0 and 10, according to the severity of their experience (subjective impact), for each of the 20 items. Thus, the similarity of the stressful event to the event experienced by the person is evaluated (1 = slightly like my experience; 10 = completely like my experience). In evaluating the scale, the total scores can be calculated and compared, or the presence of stressful life events can be determined by evaluating the results as yes/no responses. In the current study, life events that scored 10 points were determined to be deeply saddening life events.

#### 2.2.2. Hospital Anxiety and Depression Scale (HADS)

The Hospital Anxiety and Depression Scale is a 14-question scale developed to measure the level of anxiety and depression and the risk of depression and anxiety in patients with physical illness in healthcare departments [27]. The reliability and validity of the Turkish version of the scale has been improved, and a cut-off score of 10/11 was found for the anxiety subscale and 7/8 for the depression subscale [28]. The HADS is useful because it does not contain any material related to the patient’s physical state.

#### 2.2.3. The Styles of Coping with Stress Scale (CSS)

The Styles of Coping with Stress Scale (CSS) is a scale developed by Folkman [29] as part of the Ways of Coping Inventory (WCI) and was adapted and converted into a 30-item form by Şahin and Durak [30]. The items on the CSS are scored from 0 to 3 points with 4-point Likert-type responses, and separate scores are obtained for the subscales. A higher point total indicates that a style is more frequently used. The scale basically measures two main styles of coping with stress. These are “Problem-oriented/active” and “Emotion-oriented/passive” styles. Active (functional/effective) styles are represented by the “Seeking for social support”, “Optimistic approach”, and “Self-confident approach” subscales, and passive (dysfunctional/ineffective) styles are represented by the “Helpless approach” and “Submissive approach” subscales.

#### 2.2.4. Clinical Exome Sequencing

Cell-free DNA was isolated from the plasma of peripheral blood, and CES was carried out by sequencing the coding regions and splice sites of targeted genes using the Twist CES kit (covering 4909 genes (Supplementary Material S1) and 11,877,959 base dimensions (South San Francisco, CA, USA)). Cell-free DNA was extracted from peripheral blood plasma, and CES was performed by capturing the samples, which were sequenced using a DNBSEQ-G400 (MGI, Qingdao, China) after library enrichment and quality control. Variant classification and analysis were performed using the SEQ Platform (Genomize Inc., Istanbul, Turkey). FASTQ files were uploaded to the SEQ Platform and processed by aligning the reads to the human reference genome GRCh37 using the Burrows–Wheeler Aligner [31]. Aligned reads were then used for variant calling using FreeBayes [32], PCR deduplication, and in-del realignment using the proprietary algorithms by Genomize. The obtained variants were then annotated using VEP v102 [33]. ACMG pathogenicity classification was performed using Genomize’s proprietary algorithm based on the guideline published by Richards et al. [34]. Further information such as ClinVar entries and bioinformatics-based effect prediction tool scores are available in the SEQ Platform and variant frequency values are available in the SEQ cohort.

- *Classification of the genetic variants*

The genetic variants detected in patients were grouped into five classes as benign, likely benign, variant of uncertain significance (VUS), likely pathogenic, and pathogenic as defined by the Evidence-based Network for the Interpretation of Germline Mutant Alleles (ENIGMA) (https://enigmaconsortium.org/) (accessed on 12 October 2024). Our focus was on LPVs, PVs, and VUSs that could be used clinically. Variants were reported according to the nomenclature recommended by the Human Genome Variation Society (https://www.hgvs.org accessed on 12 October 2024). The clinical significance of the genetic variants found in this study was assessed with CNV analyses performed using the SEQ Platform (Genomize Inc.). Reads aligned to the human reference genome GRCh37 were assessed with the GATK gCNV tool v4.1.8.1 [35] with optimized parameters.

#### 2.2.5. Cell Culture

In this study, three different cell lines were used: human lung carcinoma cell line A549, human breast cancer cell line MCF-7, and human hypopharyngeal carcinoma cell line FaDu. The MCF-7 and FaDu cell lines were grown in DMEM (Gibco, Waltham, MA, USA) and MEM supplemented with 10% fetal bovine serum (FBS) and 1% penicillin/streptomycin (Gibco, USA), respectively. All cells were maintained at 37 °C with 5% CO_2_.

#### 2.2.6. Cell Proliferation Assay

MCF-7 and FaDu were seeded in 6-well plates as 5 × 10^5^ cells. The cells were incubated at 37 °C, with 5% CO_2_ for 24 h. Mock, WT-*ALOX12*, and WT-*FKBP5* overexpression vectors were transfected with lipofectamine 3000 reagent according to the protocol. At 0, 24, and 48 h, the cells were trypsinized, and live and dead cells were counted with a PWD C100-SE automated cell counter.

#### 2.2.7. Total RNA Isolation and Quantitative Real-Time PCR (qRT-PCR)

Total RNA from cells was isolated by using TRIzol reagent. The concentration and purity of RNA were measured by using Nanodrop (Genbiotech, Buenos Aires, Argentina) equipment. A Bio-Rad iScript cDNA synthesis kit (Bio-Rad Laboratories, Hercules, CA, USA) was used to perform cDNA synthesis. mRNA levels were measured by using a Bio-Rad iTaq Sybr Green Supermix Kit (Bio-Rad Laboratories, CA, USA). The sequences are given in Supplementary Table S1. GAPDH was used as an internal control to normalize the expression of target genes. All qRT-PCR reactions were carried out by using Bio-Rad CFX96 (Bio-Rad Laboratories, CA, USA). All reactions were performed according to the manufacturer’s protocol.

#### 2.2.8. Plasmid Transfection

*ALOX12* and *FKBP5* overexpression vectors were purchased from Origene. Overexpression vectors and the empty pcDNA3.1 vector were transiently transfected into the MCF-7 and FaDu cell lines by using lipofectamine 3000 (Thermo, Waltham, MA, USA) according to the manufacturer’s protocol. The transfection efficiency was assessed by observing the GFP of the cell lines using a fluorescence microscope.

#### 2.2.9. Invasion Assay

The invasion and migration ability of transfected cells were assessed by using BioCoat™ Matrigel^®^ Invasion Chambers (Corning, NY, ABD). A total of 2.5 × 10^4^ cells were plated in the upper chamber in a serum-free medium. The lower chamber contained 10% FBS as a chemoattractant. After 24 h of incubation in the incubator containing 5% CO_2_ at 37 °C, the non-invaded cells were removed, and the cells on the bottom surface were fixed and stained with crystal violet. Stained cells were counted under a microscope and analyzed.

#### 2.2.10. In Vivo Xenograft Models

In vivo modeling studies were carried out by outsourcing to the Boğaziçi University Experimental Animal Production and Care Unit (Vivarium). The effects of *ALOX12* and *FKBP5* genes on cancer and tumorigenesis were investigated using in vivo experiments. The FaDu and MCF-7 cell lines were transiently transfected with mock, WT-*ALOX12*, and WT-*FKBP5* overexpression vectors. The cells were collected 24 h after transfection, and 1.4 × 10^6^ cells were injected subcutaneously into the right hind leg of each mouse (6–7-week-old Balb-c nude mice) (n = 20), under anesthesia, containing 10% Matrigel. The tumor size was measured every 3 days, and the tumor volume was calculated according to the formula length × (width^2^)/2. To evaluate the tumor weight, mice injected with FaDu–mock, FaDu-*ALOX12*, FaDu-*FKBP5*, MCF-7–mock, MCF-7-*ALOX12*, and MCF-7-*FKBP5* cells were sacrificed, and the tumor weights were measured on the 48th day.

#### 2.2.11. Statistical Analysis

Patient characteristics, including gender, age, smoking history, tumor type, anxiety, depression status, and stressful life events, were tabulated according to the status of the *ALOX12* and *FKBP5* mutations. The Fisher exact test and paired sample *t* test were used for the assessment of association between the presence of *ALOX12* and *FKBP5* mutations and the patients’ characteristics and anxiety, depression status, and stressful life events. A one-way ANOVA and the paired sample *t* test were used for evaluation of the nonparametric data formed using SPSS, and a Prism *p*-value of <0.05 was considered statistically significant.

## 3. Results

### 3.1. Patient Characteristics

In this study, we aimed to determine the relationship between psychological stress and cancer. Therefore, we applied the Stressful Life Experiences Screening (SLES), the Hospital Anxiety and Depression Scale (HADS), and the Styles of Coping with Stress Scale (CSS) to the participants to examine the relationship between stress and cancer in patients referred to our psycho-oncology clinic. A total of 52 cancer patients were referred to our psycho-oncology clinic, and NGS was performed for all patients (Table 1). Out of the 52 patients, 28 were diagnosed with breast cancer, 13 with colorectal cancer, and the remaining 11 were diagnosed with other types of cancer. In total, 38 patients were under the age of 64, 32 were females, and 29 were non-smokers. Among the 52 patients, 43 had experienced a deeply saddening life event, and 9 had not. In the depression and anxiety tests, a positive score was recorded for 15/52 and 9/52 patients, respectively (Table 1). These results indicate that 43/52 patients had been exposed to a deeply saddening event in their life before cancer diagnosis.

### 3.2. Mutation Analysis

**Figure 1:**
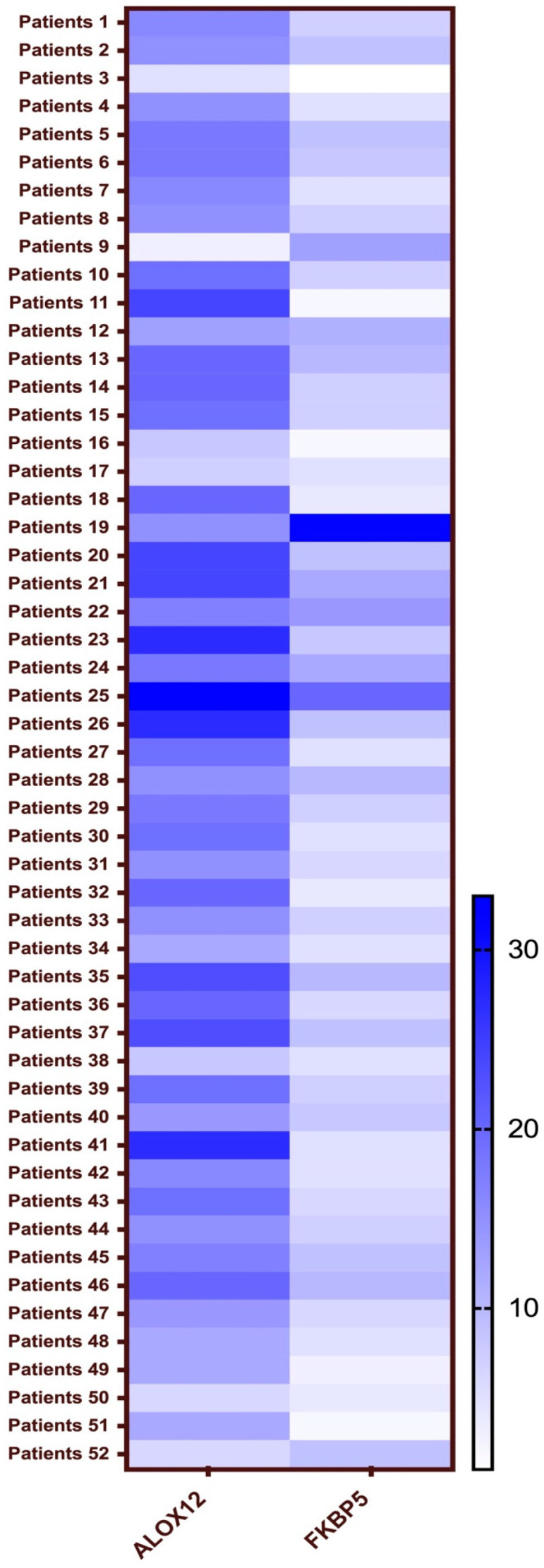
Heatmap of *ALOX12* and *FKBP5* mutations detected in cfDNA samples of patients.

We aimed to answer the question “is there any relationship between psychological stress and cancer?” Circulated tumor DNAs were isolated from these patients’ blood samples and then examined for mutations by using NGS as described in the Methods section. A stress gene panel which covers 581 stress-associated genes (Supplementary Material S1) was created for the detection of gene mutations in the patient cohort. Interestingly, NGS results showed that two genes were highly mutated in our patient cohort, which were *ALOX12* and *FKBP5* (Figure 1). We discovered that two of these mutations were commonly detected in the patient cohort positive for psychological stress, *ALOX12* c.846T > G (Asp282Glu) and *FKBP5* c.443T > G (Ile148Ser). These missense mutations have not been previously reported in the literature. The missense mutations c.443T > G (Ile148Ser) of the *FKBP5* gene were found in 13 of 43 patients who had been exposed to stressful life events. In the analysis of the *ALOX12* gene, the mutation c.846T > G (Asp282Glu) was found in 23 out of 43 patients under stress conditions, while only 1 out of 9 patients who had no exposure to stressful life events carried both mutations.

### 3.3. Relationship Between Psychological Stress and Gene Mutations

We found that patients with anxiety disorders and depressive disorders used the helpless approach more frequently as a coping style (*p* = 0.000; *p* = 0.000, respectively). Parallel to this, we found that those with anxiety disorders and depressive disorders used the self-confident approach less (*p* = 0.000; *p* = 0.000, respectively). Similarly, we found that those with anxiety disorder and depressive disorder used passive coping mechanisms more frequently (*p* = 0.006; *p* = 0.000, respectively). Anxiety levels were higher in people who had witnessed or experienced a natural disaster like a hurricane or earthquake (*p* = 0.004). Depression levels were higher in people who had seen or handled dead bodies other than at a funeral (*p* = 0.042). Other life events showed no significant differences (Table 1).

There was no significant relationship between the presence of anxiety and depression or coping styles and *ALOX12* and FKB5 mutation (*p* > 0.05). However, the important result of our study was that *ALOX12* mutation was found to be significantly higher in those with deeply saddening life events (*p* = 0.028). This shows the relationship between deeply saddening life events and mutations, independent of anxiety and depression or coping styles. When we evaluated the results according to the type of stressful life event, people who had experienced or witnessed a serious accident or injury had significantly higher *ALOX12* mutations (*p* = 0.038) (Table 1).

### 3.4. ALOX12 and FKBP5 Expression Status

**Figure 2:**
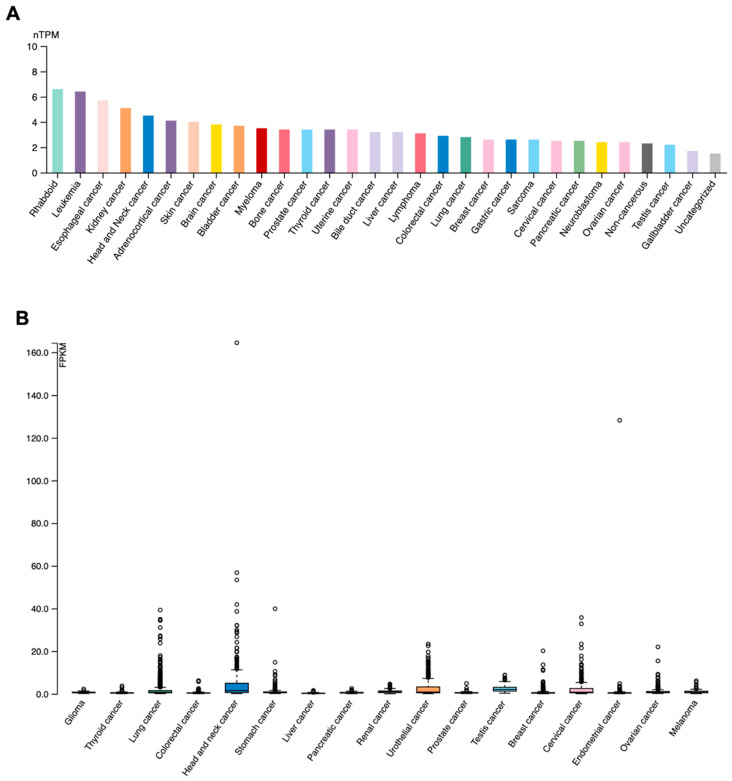

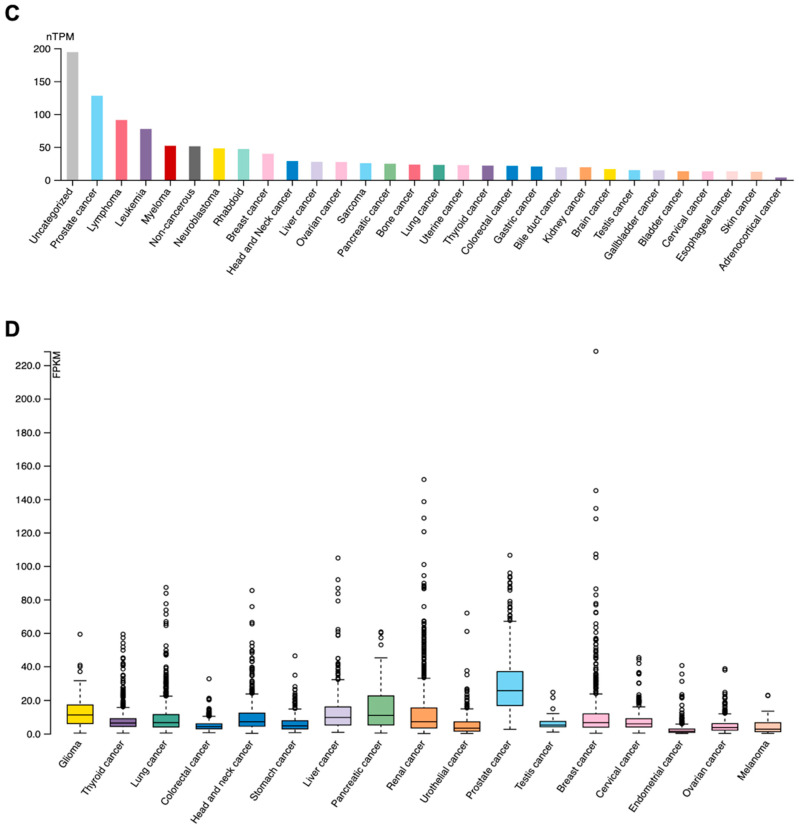
Expression status of *ALOX12* mRNA in several cancer cell lines (**A**) and in several cancer tissues (**B**). Expression status of *FKBP5* mRNA level in several cancer cell lines (**C**) and in several cancer tissues (**D**) (data from Protein Atlas).

It is known that missense mutations may affect gene expression or activity. We first searched the Protein Atlas to understand *ALOX12* and *FKBP5* expression status in cancer cell lines and cancer tissues (Figure 2A–D). The expression status of *ALOX12* and *FKBP5* genes in patients with or without cancer and several cancer cell lines was also examined using the qRT-PCR method.

**Figure 3:**
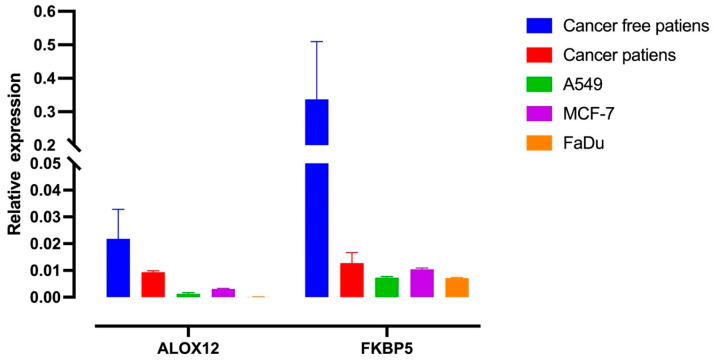
Expression levels of *ALOX12* and *FKBP5* in cancer-free patients; cancer patients; and the A549, MCF-7, and FaDu cell lines. Relative expression values of *ALOX12* and *FKBP5* in cancer-free patients, cancer patients, and cell lines were quantified with real-time PCR. Total mRNAs were isolated from blood samples of cancer and cancer-free patients, and the cDNA synthesis of the A549, MCF7, and FaDu cell lines was then performed by using a specific kit. *ALOX12* and *FKBP5* mRNA expression levels were examined by using specific primers. The expression was normalized to the expression of the house-keeping gene GAPDH. The significance was calculated using Student’s *t*-test.

**Figure 4:**
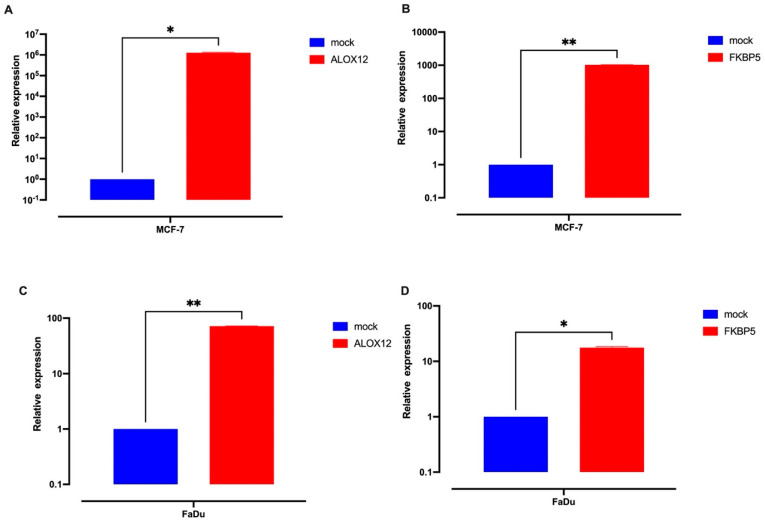
Overexpression levels of mock, *ALOX12*, and *FKBP5* in the MCF-7 cell line (**A**,**B**) and FaDu cell line (**C**,**D**). Cells were transiently transfected with mock, *ALOX12*, and *FKBP5* overexpression vectors, which also encode GFP (Green Fluorescent Protein), by using lipofectamine 3000 reagent. Forty-eight hours after the first transfection, transfection efficiency was verified and photographed under a fluorescence microscope, (Olympus Life Science, Tokyo, Japan) and total mRNAs were isolated from transgenic transfected cell lines and then cDNA synthesized by using a specific kit. *ALOX12* and *FKBP5* mRNA overexpression levels were examined by using specific primers. The expression was normalized to the expression of the house-keeping gene GAPDH. The significance was calculated using Student’s *t*-test; * *p* < 0.05 and ** *p* ≤ 0.01.

Figure 3 clearly indicates that *ALOX12* and *FKBP5* mRNA levels are downregulated in cancer cell lines and cancer tissues. It is also shown in Figure 3 that *ALOX12* and *FKBP5* mRNA levels are downregulated in cancer patients and cancer cell lines (A549, MCF-7, and FaDu) compared to cancer-free patients. Because missense mutations can inhibit gene expression, we then aimed to examine the effects of the recreated overexpression of WT-*ALOX12* and WT-*FKBP5* on cell death, cellular invasion, and tumor growth. For this approach, we used the MCF-7 and FaDu cell lines as a model because *ALOX12* and *FKBP5* mRNA expression are very low in these cell lines. Therefore, the MCF-7 and FaDu cell lines were transiently transfected with mock, WT-*ALOX12*, and WT-*FKBP5* overexpression vectors using lipofectamine 3000 reagent (Thermo, Waltham, MA, USA). Forty-eight hours after transfection, the medium was carefully removed from cells, and the mRNA was isolated for qRT-PCR reactions. As shown in Figure 4, 48 h after transient transfection, the recreated expression of *ALOX12* in the MCF-7 cell line increased approximately 106-fold, while the recreated expression of *FKBP5* increased approximately 103-fold, when compared to the mock-transfected control group. In the FaDu cell line, recreated expression levels of *ALOX12* and *FKBP5* were elevated by about 100-fold and 50-fold, respectively, compared to the mock-transfected control group.

### 3.5. Effects of WT-ALOX12 and WT-FKBP5 on Cellular Proliferation, Invasion, and Tumor Progression

**Figure 5:**
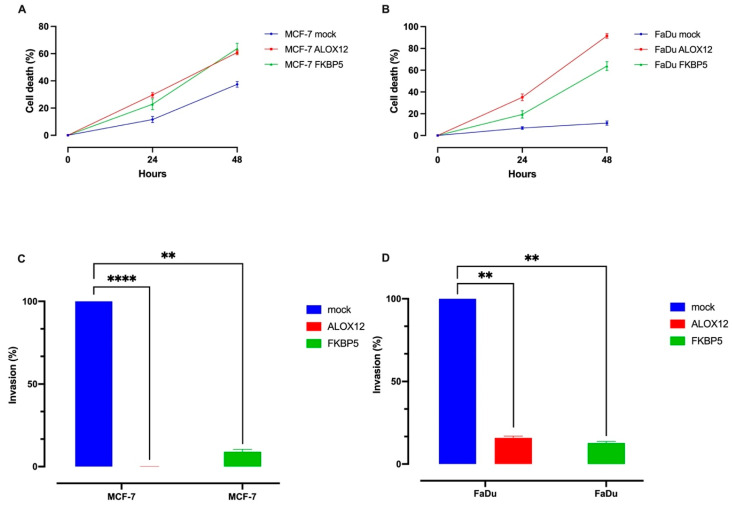
The effects of the recreated expression of *ALOX12* and *FKBP5* on the induction of cell death on MCF-7 cells (**A**) and FaDu cells (**B**) and the inhibition of the cellular invasion ability in MCF-7 (**C**) and FaDu (**D**) cell lines. The MCF-7 and *FKBP5* cell lines were transiently transfected with mock, *ALOX12*, and *FKBP5* overexpression vectors, which also encode GFP (Green Fluorescent Protein), by using lipofectamine 3000 reagent. Forty-eight hours after the first transfection, transfection efficiency was verified and photographed under a fluorescence microscope, and then 1 × 10^5^ cells were seeded into invasion chambers. The inhibitory effects of the recreated expression of the ALOX12 and FKBP5 were determined by using BD invasion chambers. The significance was calculated using Student’s *t*-test; ** *p* ≤ 0.01 and **** *p* ≤ 0.0001.

To evaluate the effects of overexpression of WT-*ALOX12* and WT-*FKBP5* on cell death, cells were trypsinized and stained with trypan blue, and the number of dead cells was automatically counted with the PWD C100-SE. In *ALOX12-* and *FKBP5*-overexpressing MCF-7 cells, the percentages of dead cells were 60% and 65%, respectively (Figure 5A). In *ALOX12-* and *FKBP5*-overexpressing FaDu cells, the percentages of dead cells were 92% and 63%, respectively (Figure 5B).

The potential inhibitory effects of overexpressing WT-*ALOX12* and WT-*FKBP5* were also examined by using Matrigel transmembrane chambers. For this approach, MCF-7 and FaDu cells were transiently transfected with mock, WT-*ALOX12*, and *FKBP5* overexpression vectors with lipofectamine 3000 reagent. Twenty-four hours after transfection, the transfection efficiency was determined under a fluorescence microscope; then, the cells were trypsinized and counted using the PWD C100-SE automated cell counter. After the Matrigel invasion chamber was prepared according to the protocol, 750 μL of chemoattractant medium was added outside the invasion chamber. A total of 500 μL of the cells in a 1 mL serum-free medium was taken and added into the invasion chamber (2.5 × 10^5^ cells). The Matrigel invasion chamber was incubated in an incubator at 37 °C, with 5% CO_2_ atmosphere for 24 h. At the end of the incubation period, non-invaded cells were removed, invaded cells were fixed according to the protocol and stained with a crystal violet dye, and then evaluated using a microscope. *ALOX12* and *FKBP5* overexpression in FaDu cells inhibited the cell invasion ability by approximately 85% compared to the mock-transfected control cells. While the invasion ability of MFC-7 cells was completely terminated in *ALOX12*-overexpressed MCF-7 cells, cellular invasion was inhibited by 90% in *FKBP5*-overexpressed MCF-7 cells when compared to mock-transfected cells (Figure 5C,D).

To evaluate the anti-metastatic ability of both the *ALOX12* and *FKBP5* genes, the FaDu and MCF-7 cell lines were transiently transfected with mock, WT-*ALOX12*, and WT-*FKBP5* overexpression vectors. The cells were collected 24 h after transfection, and 1.4 × 10^6^ cells were injected subcutaneously into the right hind leg of each mouse, under anesthesia, with 10% Matrigel. Five mice with MCF-7–mock, four mice each with MCF-7-*ALOX12* and MCF-7-*FKBP5*, three mice each with FaDu–mock, and two mice each with FaDu-*ALOX12* and FaDu-*FKBP5* were examined.

**Figure 6:**
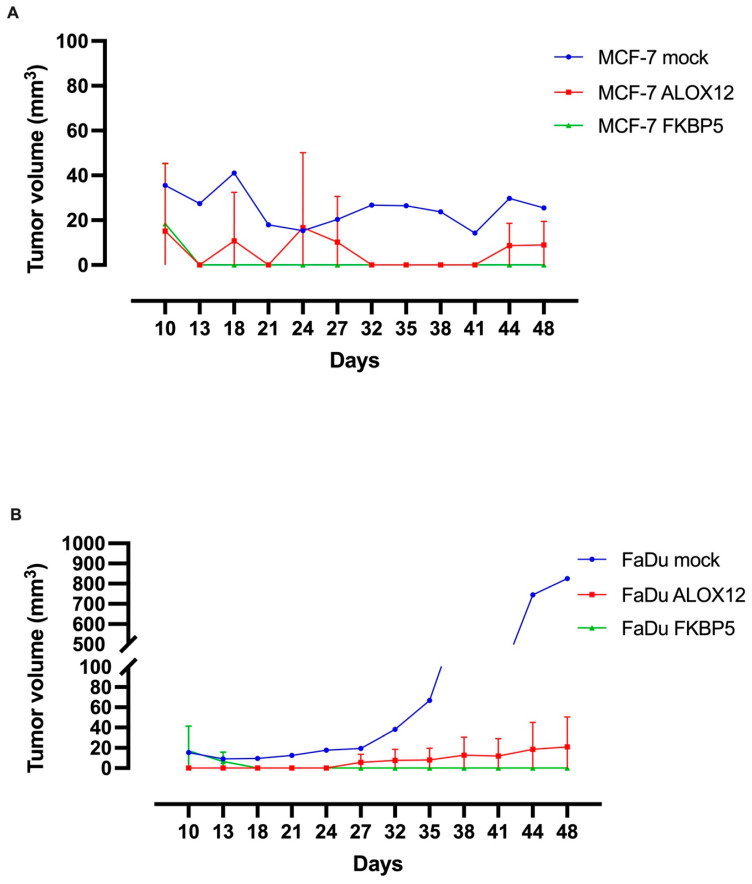

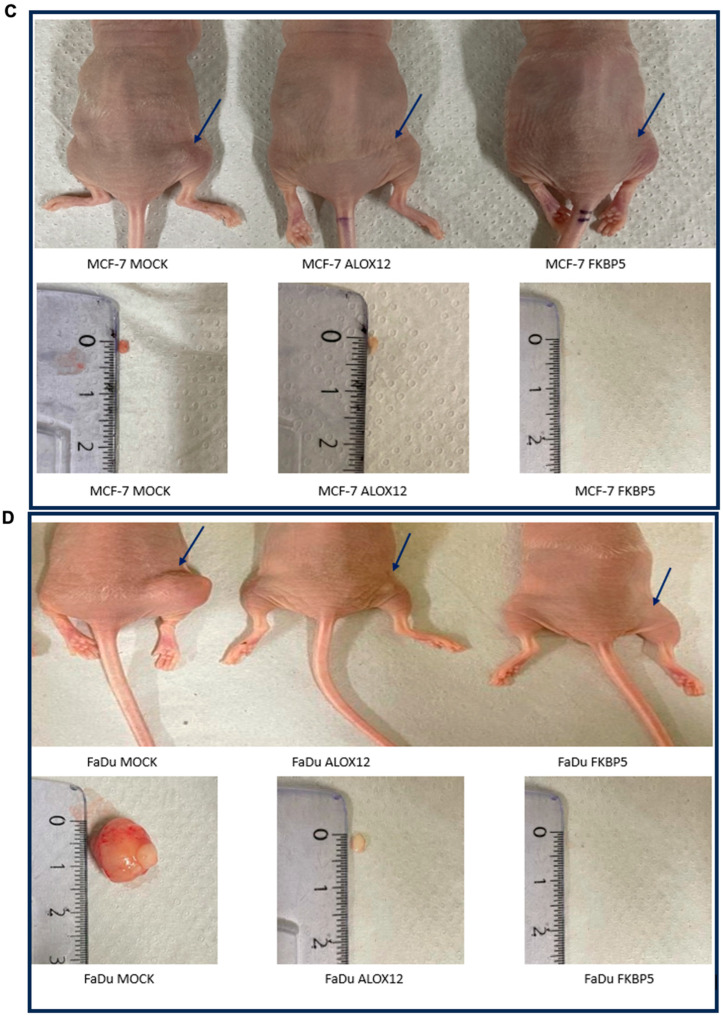

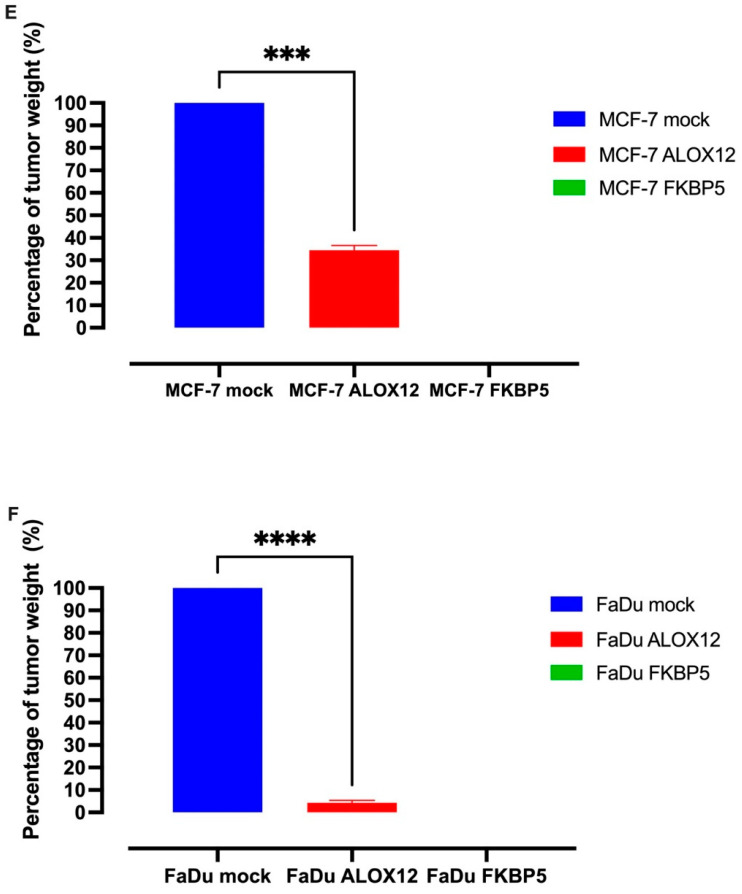
*ALOX12* and *FKBP5* overexpression were negatively correlated with tumor size. Representative mice bearing xenograft tumors derived from mock-, *ALOX12*-, and *FKBP5*-expressing MCF-7 (**A**) and FaDu (**B**) cell lines (*p* < 0.05, two-way analysis of variance) when compared to mock expressing cells. Comparison of tumors excised from xenograft mice at 48 days after inoculation (**C**,**D**). Data are mean ± SD (N = 3). *p* < 0.05, compared to the mock-transfected control. MCF-7–mock, MCF-7-ALOX12, MCF-7-FKBP5, FaDu–mock, FaDu-ALOX12, and FaDu-FKBP5 injected mice were sacrificed, and tumor weight was measured at the 48th day (**E**,**F**). The significance was calculated using Student’s *t*-test; *** *p* ≤ 0.001 and **** *p* ≤ 0.0001.

Starting on the third day after injection, the tumor volume formed at the injection site was measured at intervals of 3 days, and the data were recorded for mice injected with FaDu–mock, FaDu-ALOX12, FaDu-FKBP5, MCF-7–mock, MCF-7-ALOX12, and MCF-7-FKBP5 cells. Tumor formation was observed on the 10th day in the FaDu–mock and FaDuFKBP5 groups, while in the FaDu-ALOX12 group it started on the 27th day. The tumor volume was 3-fold smaller on the 27th day, 8-fold smaller on the 35th day, and 37-fold smaller on the 48th day in the FaDu-ALOX12 group compared to FaDu–mock group on the same days. Despite this, the volume of the tumor in the FaDu–mock group and FaDu-FKBP5 group was almost the same size on the 10th day and started to decrease on the 13th day, and the tumor had disappeared by the 18th day and was not observed in the remaining days. Tumor formation occurred on the 10th day in the MCF-7–mock group, MCF-7-ALOX12 group, and MCF-7-FKBP5 group. In the MCF-7-ALOX12 group, the tumor volume was approximately 2-fold smaller compared to the MCF-7–mock group on the 27th day and 48th day. Tumors formed on the 10th day but started to decrease and disappeared on the 13th day in the MCF-7-FKBP5 group (Figure 6A–D).

FaDu–mock, FaDu-ALOX12, FaDu-FKBP5, MCF-7–mock, MCF-7-ALOX12, and MCF-7-FKBP5 mice were sacrificed, and the tumor weights were measured on the 48th day. The recreated WT-ALOX12 overexpression decreased the tumor weight in both the FaDu and MCF-7 groups. Tumor formation was not detected in either of the FaDu-FKBP5 and MCF-7-FKBP5 groups on the day of sacrifice (Figure 6E,F). All of the animals were also evaluated for metastasis, but no evidence of metastasis was found.

### 3.6. ALOX12 and FKBP5 Expression Induce LC3b Expression and Autophagy in Both MCF7 and FaDu Cell Lines

**Figure 7:**
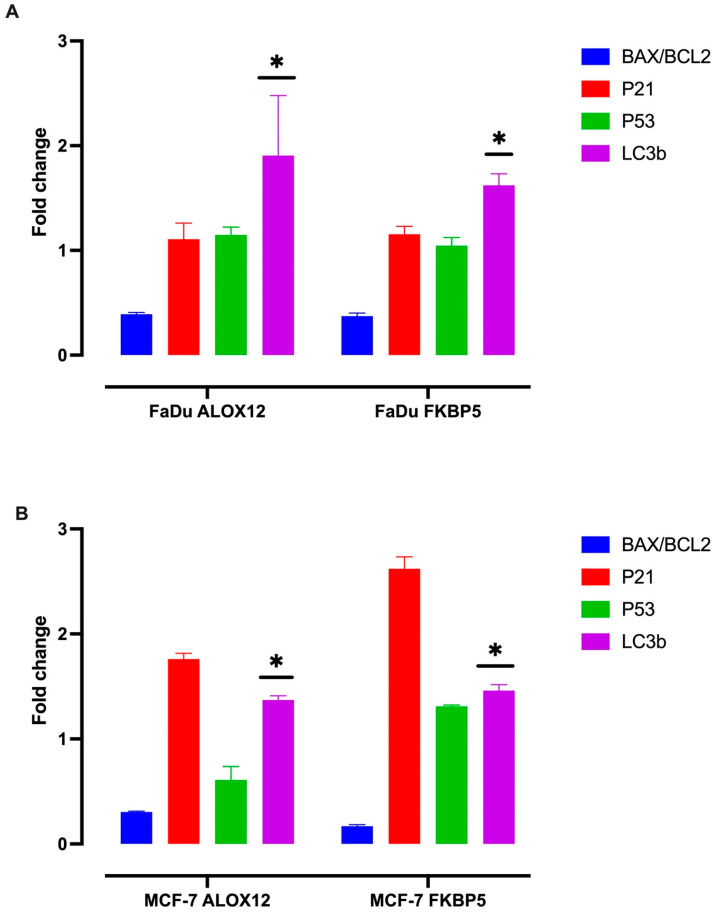
The effects of the recreated expression of *ALOX12* and *FKBP5* genes on *Bax*/*Bcl2* ratio, *p21*, *p53*, and *LC3b* expression in FaDu cells (**A**) and MCF7 cells (**B**) * (*p* ˂ 0.005).

Because our results indicated that both *ALOX12* and *FKBP5* gene expression induced cellular death and inhibited the tumor growth and invasion ability of cancer cells, we aimed to examine the molecular mechanism of these antitumor effects. Therefore, we transgenically transfected the MCf7 and FaDu cell lines with mock, WT-*ALOX12*, and Wt-*FKBP5* genes. After 48 h, lysed cells were isolated, and the expression status of target genes was examined as described in the Methods section. Figure 5 clearly indicates that WT-*ALOX12* and *FKBP5* gene expression decrease *Bax/bcl2* expression with no effect on *p53* expression but increase the expression level of autophagy marker *LC3b* expression in both cell lines (*p* ˂ 0.005) (Figure 7A,B).

## 4. Discussion

Many researchers have found that chronic stress can wear down our body’s defenses, lower our immune response, and make us more vulnerable to most sicknesses, including cancer [36]. Chronic stress also increases the production of certain growth factors in patients’ blood. This may increase the development of cancerous tumors [37]. Previously, researchers have reported that stress can modify DNA methylation, causing DNA polymorphism, which may alter gene expression and thereby contribute to disease phenotypes. There is evidence linking stress exposure to increased cancer risk via epigenetic mechanisms. In a 2008 meta-analysis of 142 prospective studies among people in Asia, Australia, Europe, and America, stress was associated with a higher incidence of lung cancer [38], and in 2019, a meta-analysis of 9 observational studies in Europe and North America found an association between work stress and the risk of lung, colorectal, and esophageal cancers [39].

However, there is still missing evidence on whether extremely stressful life events could create somatic mutations in genes that induce tumorigenesis. In this study, we aimed to explain the relationship between psychological stress and cancer. Therefore, we focused on cancer patients who had experienced stressful life events before being diagnosed with cancer. We evaluated cancer patients referred to our psycho-oncology polyclinic for stressful life experiences, anxiety/depression levels, and coping styles with scales. A structured clinical interview (SCID-5) was also applied to determine the patients’ state of mind. After this evaluation, cfDNA was isolated from 52 patients, and CES analysis was performed using the MCI platform. Surprisingly, we found that two genes were highly mutated in the deeply saddening life events-positive patient cohort for traumatic life events when compared to the negative patient cohort. These genes are *ALOX12* and *FKBP5*. We found the c.846T>G (Asp282Glu) missense mutation on *ALOX12* and the c.443T>G (Ile148Ser) missense mutation on the *FKBP5* gene. Moreover, these mutations are novel missense mutations that have not been reported in the literature. Chu et al. (2019) showed that missense mutations of *ALOX12* abrogate its ability to oxygenate polyunsaturated fatty acids and to induce *p53*-mediated ferroptosis [40]. This research clearly showed that some *ALOX12* missense mutations can strongly inhibit the *ALOX12* antitumor effect. This finding supports our results because the new missense mutations in *ALOX12* and *FKBP5* genes in our study inhibited an antitumor effect, possibly via the downregulation of gene expression. This is because, in cancer patients, the A549, MCF-7, and FaDu cell lines express lower levels of *ALOX12* and *FKBP5* mRNAs when compared to the cancer-free patient group, as indicated in our qRT-PCR results. The Protein Atlas also shows that *ALOX12* and *FKBP5* have low expression both in cancer tissue and cancer cell lines. Moreover, Chu et al. reported that *ALOX12* expression is downregulated in many cancer types, including cervical carcinoma, head and neck carcinoma, esophageal carcinoma, and acute myeloid leukemia. These researchers also reported that some missense mutations of *ALOX12* lead to the downregulation of its gene expression and the enzyme activation of the *ALOX12* gene [40]. These results are in alignment with our findings.

Falcinelli et al. reported that psychological stress can induce cancer initiation via the inhibition of the DNA repair mechanism and inducing of mutations in DNA [41]. Our results are in alignment with this report because we found *ALOX12* and *FKBP5* somatic mutations in cancer patients who had experienced traumatic life events. These *ALOX12* and *FKBP5* somatic missense mutations may be induced via the psychological stress experienced by these patients, which may also contribute to cancer initiation and progression. Even though our study covers almost all cancer types, it still has some limitations. Firstly, the patient cohort was 52 patients, and secondly, cancer types were not completely homogeneous in the patient cohort (28 cases of breast cancer, 13 colorectal cancer, 5 rectum cancer, 2 prostate cancer, 1 lung cancer, 1 nasopharynx cancer, and 1 non-Hodgkin’s lymphoma). Therefore, future studies with a larger patient cohort size with homogenous cancer types must be performed.

Since Chu et al. describe that the ALOX12 antitumor effect can be inhibited by missense mutations [39], we aimed next to examine the effects of the recreated overexpression of WT-ALOX12 and WT-FKBP5 on cell death, cellular invasion, and tumor growth. When we transfected MCF-7 and FaDu cells with WT-*ALOX12* and WT-*FKBP5* genes, the recreated overexpression of *ALOX12* and *FKBP5* led to the induction of cellular death, inhibition of cellular invasion abilities, and strong inhibition of xenograft tumor growth. The relationship between cancer and *FKBP5* and *ALOX12* gene expression has been evaluated in several studies, and the results support our findings. Pei et al. indicate that *FKBP5* expression is dramatically downregulated in pancreatic cancer [42], and Cugliari reported that *FKBP5* can be a missing key element between psychological stress and cancer [43]. Chu et al. indicate that *ALOX12* expression is also dramatically downregulated in several cancers, including cervical cancer, head and neck cancer, and acute myeloid leukemia [40]. Our results clearly indicate that WT-*ALOX12* and *FKBP5* gene expression induce *Lc3b* expression, decrease *Bax/bcl2* ratio, and have no effect on *p53* expression. This means that the WT-*ALOX12* and *FKBP5* antitumor effects derive from the induction of autophagy, and a higher level of somatic mutations accumulated on *FKBP5* and *ALOX12* genes in cancer patients inhibit *ALOX12* and *FKBP5* gene expression and increase cancer progression in cancer patients who were exposed to stressful life events.

Coping mechanisms are important in the fight against cancer. A study of the literature showed that young breast cancer survivors relied heavily on the support of family and friends, and those with greater reliance on emotional support from family were less likely to have anxiety after 2 years [44]. In another study, it was remarked that anxiety, depression, and fear of cancer recurrence dominated patients’ emotional concerns, and Asian breast cancer patients coped with the help of social support, positive reappraisal, and faith in religion. Consistent with the relevant literature, we found that those with anxiety disorder and depressive disorder use passive coping mechanisms more frequently. We also found that anxiety and depression levels were associated with stressful life events.

The findings regarding whether stressful life events increase cancer risk or progression are insufficient. Researchers have begun to answer key questions about how stressful life events and the negative emotions they generate can impact cancer initiation, progression, and survivorship. Molecular studies have identified biological processes that could potentially mediate the role of stressful life experiences in modulating physiological processes related to cancer development and indicated that chronic depression and a lack of social support might serve as risk factors for cancer development and progression [41,45,46,47]. Chida et al. associated stressful life events with poorer survival among samples of patients with cancer, as well as with higher mortality rates due to cancer in population samples [38]. Duijts et al. identified an association between the death of a spouse and breast cancer risk. They indicate that the categories death of a spouse and death of a relative or friend appear to be more influential than the categories change in marital status and change in financial status [12]. We found that in cancer patients, anxiety levels were higher in patients who had witnessed or experienced a natural disaster like a hurricane or earthquake, and depression levels were higher in patients who had seen or handled dead bodies other than at a funeral. The ALOX12 mutation rate was significantly higher in those who had experienced traumatic life events. According to the analysis of stressful life event types, patients who had experienced or witnessed a serious accident or injury had significantly higher rates of *ALOX12* mutation.

## 5. Conclusions

In this study, we tried to answer the question “*is there any interaction between psychological stress and cancer at the gene level?*” Our NGS results showed that a higher level of mutations accumulated on the *FKBP5* and *ALOX12* genes in cancer patients who were exposed to stressful life events. Among these mutations, two new missense mutations of c.846T>G (Asp282Glu) and c.443T>G (Ile148Ser) were observed in *ALOX12* and *FKBP5*, respectively. The expression status of *ALOX12* and *FKBP5* genes in patients with or without cancer and several cancer cell lines demonstrated that both *ALOX12* and *FKBP5* mRNA levels were downregulated in cancer patients and cancer cell lines (A549, MCF-7, and FaDu). To examine whether the recreated expression of *ALOX12* and *FKBP5* has any effect on cancer development, WT-*ALOX12* and WT-*FKBP5* were overexpressed in the MCF-7 and FaDu cell lines. The recreated overexpression of WT-*ALOX12* and WT-*FKBP5* significantly inhibited cellular growth and cellular invasion. Moreover, the overexpression of these genes in the same cell lines dramatically suppressed tumor growth in a xenograft model. Our results clearly indicate that psychological stress may induce cancer development via increased somatic missense mutations in *ALOX12* and *FKBP5* genes.

## Figures and Tables

**Table 1:** Correlation between *FKBP5* and *ALOX12* mutations and clinicopathological characteristics in patients.

			*FKBP5* Mutation (%)			*ALOX12* Mutation (%)		
Clinicopathological Characteristic			+	−		+	−	
	Subset	Number of Patients (n = 52)	(n = 14)	(n = 38)	*p* Value	(n = 24)	(n = 28)	*p* Value
Age	<64	38	7 (18.42)	31 (81.58)	0.035 ^a^	14 (36.84)	24 (63.16)	0.033 ^a^
	≥64	14	7 (50)	7 (50)		10 (71.43)	4 (28.57)	
Gender	Male	20	4 (20)	16 (80)	0.524 ^a^	7 (35)	13 (65)	0.258 ^a^
	Female	32	10 (31.25)	22 (68.75)		17 (53.13)	15 (46.88)	
Smoking history	Smoker	19	3 (15.79)	16 (84.21)	0.156 ^a^	8 (42.11)	11 (57.89)	0.359 ^a^
	Non-smoker	33	11 (33.33)	22 (66.67)		16 (48.48)	17 (51.52)	
Stressful life events	Stressful	43	13 (30.23)	30 (69.77)	<0.001 ^b^	23 (53.49)	20 (46.51)	<0.001 ^b^
	Non-stressful	9	1 (11.11)	8 (88.89)		1 (11.11)	8 (88.89)	
Depression	Yes	15	5 (33.33)	10 (66.67)	0.821 ^b^	8 (53.33)	7 (46.67)	0.06 ^b^
	No	37	9 (24.32)	28 (75.68)		16 (43.24)	21 (56.76)	
Anxiety	Yes	9	2 (22.22)	7 (77.78)	0.255 ^b^	4 (44.44)	5 (55.56)	0.002 ^b^
	No	43	12 (27.91)	31 (72.09)		20 (46.51)	23 (53.49)	
Cancer Type	Breast Cancer	28	10 (35.71)	18 (64.29)	0.016 ^a^	15 (53.57)	13 (46.43)	0.056 ^a^
	Colorectal Cancer	13	1 (7.69)	12 (92.31)		4 (30.77)	9 (69.23)	
	Rectum Cancer	5	1 (20)	4 (80)		1 (20)	4 (80)	
	Prostate Cancer	2	1 (50)	1 (50)		2 (100)	-	
	Pancreatic Cancer	1	-	1 (100)		-	1 (100)	
	Lung Cancer	1	1 (100)	-		1 (100)	-	
	Nasopharynx Cancer	1	-	1 (100)		-	1 (100)	
	Non-Hodgkin’s lymphoma	1	-	1 (100)		1 (100)	-	

^a^ Fisher’s exact test; ^b^ Paired sample *t* test.

## Data Availability

Data are available from the authors upon reasonable request.

## Supplementary Materials

The following supporting information can be downloaded at: https://www.mdpi.com/article/10.3390/genes15121531/s1, Table S1: The primer sequences of *ALOX12*, *FKBP5*, *GAPDH, P21*, *P53*, *Bax*, *Bcl2*, and *LC3b* genes; Supplementary Materials S1: Twist CES genes. Supplementary Materials S2: Stress-related genes panel.
