# Supplementary material for: Deeply Saddening Life Events Play a Carcinogenic Role by Inducing Mutations in ALOX12 and FKBP5 Genes
Source: Genes (Basel). 2024 Nov 28;15(12):1531. doi: 10.3390/genes15121531 (PMC11675945; doi:10.3390/genes15121531)
Supplement: Supplementary file 1 [file genes-15-01531-s001.zip › genes-3283330-supplementary.pdf]

Accession Number	Genes	Primer's sequencing
NM_000697 Location: intron	ALOX12 Sense	5'-CCTCGTTATGCTGAAGATGGAGC-3'
	ALOX12 Antisense	5'-CCGTTTCAGGACCCAGGCTTTA-3'
NC_004117 Location: intron	FKBP5 Sense	5'- GCGAAGGAGAAGACCACGACAT-3'
	FKBP5 Antisense	5'-AAACCTCTCCGTCCCTTCGGAT-3'
NM_004324	BAX Sense	5'-TCAGGATGCGTCCACCAAGAAG-3'
	BAX Antisense	5'-TGTGTCCACGGCGGCAATCATC-3'
NM_000633	BCL2 Sense	5'-ATCGCCCTGTGGATGACTGAGT-3'
	BCL2 Antisense	5'-GCCAGGAGAAATCAAACAGAGGC-3'
NM_000389	P21 Sense	5'-AGGTGGACCTGGAGACTCTCAG-3'
	P21 Antisense	5'-TCCTCTTGGAGAAGATCAGCCG-3'
NM_000546	P53 Sense	5'-CCTCAGCATCTTATCCGAGTGG-3'
	P53 Antisense	5'-TGGATGGTGGTACAGTCAGAGC-3'
NM_022818	LC3b Sense	5'-GAGAAGCAGCTTCCTGTTCTGG-3'
	LC3b Antisense	5'-GTGTCCGTTCACCAACAGGAAG-3'
NM_002046	GAPDH Sense	5'- GTCTCCTCTGACTTCAACAGCG -3'
	GAPDH Antisense	5'- ACCACCCTGTTGCTGTAGCCAA -3'

Table 1. The Primer sequences of *ALOX12*, *FKBP5*, *GAPDH*, *P21*, *P53*, *Bax*, *Bcl2* and *LC3b* genes.

Supplementary material 1:
TWIST CES GENES

A2M
A4GALT
A4GNT
AAAS
AADAC
AADACL2
AAGAB
AANAT
AARS2
AASS
ABAT
ABCA1
ABCA2
ABCA3
ABCA4
ABCA10
ABCA12
ABCA13
ABCB1
ABCB4
ABCB6
ABCB7
ABCB11
ABCC1
ABCC2
ABCC3
ABCC4
ABCC6
ABCC8
ABCC9
ABCC11
ABCC12
ABCD1
ABCD3
ABCD4
ABCG2
ABCG5
ABCG8
ABHD5
ABHD12
ABI3BP
ABL1

ABL2
ABO
ACACB
ACAD8
ACAD9
ACAD10
ACAD11
ACADL
ACADM
ACADS
ACADSB
ACADVL
ACAN
ACAT1
ACAT2
ACBD5
ACCS
ACE
ACHE
ACKR1
ACLY
ACO2
ACOX1
ACP1
ACP2
ACP5
ACSF3
ACSL4
ACSL5
ACSL6
ACSM3
ACTA1
ACTA2
ACTB
ACTC1
ACTG1
ACTN1
ACTN2
ACTN4
ACVR1
ACVR1B
ACVR1C
ACVR2A
ACVR2B

ACVRL1
ACY1
ADA
ADAM7
ADAM9
ADAM10
ADAM12
ADAM17
ADAM19
ADAM22
ADAM23
ADAM33
ADAMTS1
ADAMTS2
ADAMTS10
ADAMTS13
ADAMTS17
ADAMTS18
ADAMTS19
ADAMTSL2
ADAMTSL3
ADAMTSL4
ADAR
ADARB1
ADCY3
ADCY5
ADCY6
ADCY9
ADCY10
ADD1
ADD2
ADH1A
ADH1B
ADH1C
ADH4
ADH7
ADIPOQ
ADIPOR1
ADK
ADM
ADORA2A
ADRA1A
ADRA2A
ADRA2C

ADRB1
ADRB2
ADRB3
ADSL
ADTRP
AFF2
AFF3
AFG3L2
AFP
AGA
AGGF1
AGK
AGL
AGMO
AGO1
AGPAT2
AGPS
AGRN
AGRP
AGT
AGTPBP1
AGTR1
AGTR2
AGXT
AGXT2
AHCY
AHI1
AHR
AHSG
AHSP
AICDA
AIFM1
AIMP1
AIP
AIPL1
AIRE
AK1
AK2
AK7
AK8
AKAP2
AKAP9
AKAP10
AKR1B1

AKR1C1
AKR1C2
AKR1C3
AKR1C4
AKR1D1
AKR7A2
AKR7A3
AKT1
AKT2
AKT3
ALAD
ALAS2
ALB
ALDH1A1
ALDH1A2
ALDH2
ALDH3A2
ALDH4A1
ALDH5A1
ALDH6A1
ALDH7A1
ALDH16A1
ALDH18A1
ALDOA
ALDOB
ALG1
ALG1L
ALG1L2
ALG2
ALG3
ALG5
ALG6
ALG8
ALG9
ALG11
ALG12
ALG13
ALG14
ALK
ALMS1
ALOX5
ALOX5AP
ALOX12
ALOX12B

ALOX15
ALOXE3
ALPL
ALS2
ALS2CL
ALX1
ALX3
ALX4
AMACR
AMBN
AMELX
AMELY
AMER1
AMH
AMHR2
AMN
AMPD1
AMPD3
AMT
ANG
ANGPT2
ANGPTL3
ANGPTL4
ANGPTL5
ANGPTL6
ANK1
ANK2
ANK3
ANKH
ANKK1
ANKRD1
ANKRD11
ANKRD26
ANKS1A
ANKS6
ANO3
ANO5
ANO6
ANO7
ANO10
ANTXR1
ANTXR2
ANXA5
AOAH

AP1S1
AP1S2
AP3B1
AP3B2
AP3D1
AP4B1
AP4E1
AP4M1
AP4S1
AP5Z1
APAF1
APBA2
APBB1IP
APBB2
APC
APCDD1
APEX1
APOA1
APOA2
APOA4
APOA5
APOB
APOBEC1
APOBEC3H
APOC2
APOC4
APOD
APOE
APOH
APOL1
APOL3
APOL4
APOOL
APP
APRT
APTX
AQP1
AQP2
AQP3
AQP4
AQP5
AQP7
AR
ARF4

ARFGEF2
ARG1
ARHGAP6
ARHGAP9
ARHGAP18
ARHGAP24
ARHGAP26
ARHGAP31
ARHGDIA
ARHGEF6
ARHGEF7
ARHGEF9
ARHGEF10
ARHGEF12
ARID1A
ARID1B
ARID4A
ARID4B
ARL3
ARL6
ARL13B
ARL14EP
ARMS2
ARSA
ARSB
ART4
ARX
AS3MT
ASAH1
ASB10
ASCC1
ASCC3
ASCL1
ASIC3
ASIP
ASL
ASMT
ASNS
ASPA
ASPM
ASPN
ASPRV1
ASS1
ASTE1

ASTN2
ASXL1
ATCAY
ATF3
ATF6
ATG7
ATG9B
ATG16L1
ATIC
ATL1
ATM
ATN1
ATOH7
ATP1A2
ATP1A3
ATP1A4
ATP1B1
ATP2A1
ATP2A2
ATP2A3
ATP2B2
ATP2B3
ATP2B4
ATP2C1
ATP6AP2
ATP6V1B1
ATP6V0A2
ATP6V0A4
ATP7A
ATP7B
ATP8A2
ATP8B1
ATP10A
ATP10D
ATP13A2
ATP13A4
ATPAF2
ATR
ATRNL1
ATRX
ATXN1
ATXN2
ATXN3
ATXN3L

ATXN7
ATXN10
AUH
AURKA
AURKC
AUTS2
AVP
AVPR1A
AVPR2
AXDND1
AXIN1
AXIN2
AXL
AZIN2
B2M
B3GALNT1
B3GALNT2
B3GALT1
B3GALT2
B3GALT5
B3GALT6
B3GAT1
B3GAT2
B3GAT3
B3GNT2
B3GNT3
B3GNT4
B3GNT5
B3GNT6
B3GNT7
B3GNT8
B4GALNT1
B4GALNT2
B4GALNT3
B4GALNT4
B4GALT1
B4GALT2
B4GALT3
B4GALT4
B4GALT5
B4GALT6
B4GALT7
B9D1
B9D2

BAAT
BAG3
BANF1
BANK1
BAP1
BARD1
BARX2
BAX
BAZ1B
BBS1
BBS2
BBS4
BBS5
BBS7
BBS9
BBS10
BBS12
BCAM
BCAT2
BCHE
BCKDHA
BCKDHB
BCKDK
BCL2
BCL2L1
BCL2L2
BCL2L2-PABPN1
BCL2L11
BCL6
BCL9
BCL10
BCL11A
BCO1
BCOR
BCORL1
BCR
BCS1L
BDNF
BEAN1
BEST1
BFSP1
BFSP2
BGN
BHLHA9

BHLHE41
BICC1
BIN1
BIVM-ERCC5
BLK
BLM
BLMH
BLNK
BLOC1S5
BLOC1S6
BLVRA
BMI1
BMP1
BMP2
BMP4
BMP5
BMP7
BMP15
BMPER
BMPR1A
BMPR1B
BMPR2
BNC2
BOC
BOLA3
BPGM
BRAF
BRAT1
BRCA1
BRCA2
BRCC3
BRD1
BRD2
BRF1
BRIP1
BRSK2
BRWD1
BRWD3
BSCL2
BSG
BSND
BTBD9
BTD
BTK

BUB1
BUB1B
C1GALT1
C1GALT1C1
C1QA
C1QB
C1QC
C1QTNF5
C1R
C1S
C2orf42
C3
C3AR1
C4orf19
C5
C5AR2
C6
C7
C8A
C8B
C8orf37
C9
C9orf72
C9orf139
C10orf55
C10orf105
C12orf29
C12orf57
C15orf62
C17orf107
C21orf91
CA1
CA2
CA4
CA8
CA12
CABIN1
CABP2
CABP4
CACNA1A
CACNA1C
CACNA1D
CACNA1E
CACNA1F

CACNA1G
CACNA1H
CACNA1I
CACNA1S
CACNA2D1
CACNA2D2
CACNA2D3
CACNA2D4
CACNB2
CACNB4
CACNG2
CACNG3
CACNG4
CADM1
CADPS2
CALCA
CALCB
CALCR
CALCRL
CALHM1
CALM1
CALM3
CALR
CALR3
CALU
CAMK1
CAMK4
CAMKMT
CAMSAP2
CAMTA1
CANT1
CAPN3
CAPN10
CARD9
CARD11
CARD14
CARD16
CARTPT
CASK
CASP1
CASP2
CASP8
CASP10
CASP12

CASQ2
CASR
CAST
CAT
CATSPER1
CATSPER2
CATSPER3
CATSPER4
CAV1
CAV3
CBFB
CBL
CBLB
CBR1
CBR3
CBS
CBX2
CBX4
CBY1
CC2D1A
CC2D2A
CCBE1
CCDC8
CCDC12
CCDC14
CCDC22
CCDC28B
CCDC40
CCDC50
CCDC66
CCDC78
CCDC88C
CCDC103
CCDC107
CCDC127
CCDC140
CCKAR
CCKBR
CCL2
CCL11
CCM2
CCND1
CCR2
CCR3

CCR5
CCR7
CCT5
CD2AP
CD3D
CD3E
CD3G
CD4
CD5
CD8A
CD19
CD27
CD36
CD38
CD40
CD40LG
CD44
CD46
CD55
CD59
CD74
CD79A
CD79B
CD80
CD81
CD86
CD96
CD109
CD151
CD177
CD200
CD207
CD209
CD244
CD247
CD320
CDA
CDAN1
CDC6
CDC42
CDC42BPB
CDC73
CDCA3
CDCA7L

CDH1
CDH3
CDH5
CDH13
CDH15
CDH23
CDHR1
CDK4
CDK5R1
CDK5RAP2
CDK6
CDK7
CDK11A
CDK11B
CDK16
CDKAL1
CDKL3
CDKL5
CDKN1A
CDKN1B
CDKN1C
CDKN2A
CDKN2B
CDKN2C
CDON
CDT1
CDX2
CDYL
CEACAM16
CEBPA
CEBPE
CECR2
CEL
CELSR1
CELSR2
CEMIP
CENPJ
CENPO
CENPP
CEP41
CEP57
CEP63
CEP85L
CEP135

CEP152
CEP164
CEP290
CERKL
CERS1
CERS6
CES1
CES2
CETP
CFAP53
CFAP57
CFD
CFH
CFHR1
CFHR2
CFHR5
CFI
CFL2
CFP
CFTR
CGB1
CGB2
CGB5
CGB7
CGB8
CGREF1
CHAT
CHD1L
CHD2
CHD3
CHD6
CHD7
CHD8
CHEK2
CHGA
CHI3L1
CHIA
CHIC2
CHIT1
CHKB
CHL1
CHM
CHMP1A
CHMP2B

CHMP4B
CHN1
CHPF
CHPF2
CHPT1
CHRD
CHRDL1
CHRFAM7A
CHRM1
CHRM2
CHRM3
CHRNA1
CHRNA2
CHRNA3
CHRNA4
CHRNA5
CHRNA7
CHRNA9
CHRNB1
CHRNB2
CHRNB4
CHRND
CHRNE
CHRNG
CHST3
CHST5
CHST6
CHST7
CHST8
CHST14
CHSY1
CHSY3
CHUK
CHURC1-FNTB
CIAPIN1
CIB2
CIC
CIDEC
CIITA
CILP
CISD2
CISH
CITED2
CIZ1

CKM
CLCC1
CLCF1
CLCN1
CLCN2
CLCN3
CLCN4
CLCN5
CLCN6
CLCN7
CLCNKA
CLCNKB
CLDN1
CLDN14
CLDN16
CLDN19
CLEC3B
CLEC4M
CLEC7A
CLEC10A
CLIC2
CLIP2
CLK2
CLMP
CLN5
CLN6
CLN8
CLOCK
CLPTM1
CLPTM1L
CLRN1
CLTCL1
CLU
CMPK1
CNBP
CNGA1
CNGA3
CNGB1
CNGB3
CNKSR1
CNKSR2
CNNM2
CNNM3
CNNM4

CNOT4
CNPY3
CNR1
CNR2
CNTF
CNTN1
CNTN4
CNTNAP2
CNTNAP4
COA5
COCH
COG1
COG2
COG3
COG4
COG5
COG6
COG7
COG8
COL1A1
COL1A2
COL2A1
COL3A1
COL4A1
COL4A2
COL4A3
COL4A4
COL4A5
COL4A6
COL5A1
COL5A2
COL6A1
COL6A2
COL6A3
COL7A1
COL8A2
COL9A1
COL9A2
COL9A3
COL10A1
COL11A1
COL11A2
COL17A1
COL18A1

COL21A1
COLEC11
COLQ
COMMD1
COMMD3-BMI1
COMP
COMT
COPA
COQ2
COQ5
COQ6
COQ9
CORIN
CORO1A
COX4I1
COX4I2
COX6B1
COX7B
COX10
COX14
COX15
CP
CPA6
CPB2
CPE
CPLX2
CPN1
CPOX
CPS1
CPT1A
CPT2
CPZ
CR1
CR1L
CR2
CRADD
CRB1
CRBN
CREB1
CREB3L3
CREBBP
CRELD1
CRISP2
CRKL

CRLF1
CRP
CRTAP
CRX
CRYAA
CRYAB
CRYBA1
CRYBA4
CRYBB1
CRYBB2
CRYBB3
CRYGA
CRYGB
CRYGC
CRYGD
CRYGS
CRYM
CSAG1
CSDE1
CSF1
CSF1R
CSF2
CSF2RA
CSF2RB
CSF3R
CSGALNACT1
CSH1
CSH2
CSHL1
CSMD1
CSMD3
CSNK1D
CSNK1G1
CSNK2A2
CSRP3
CST3
CST6
CSTA
CSTB
CSTF2
CSTF2T
CTC1
CTDP1
CTF1

CTH
CTHRC1
CTLA4
CTNNA2
CTNNA3
CTNNB1
CTNND2
CTNS
CTRC
CTSA
CTSB
CTSC
CTSD
CTSG
CTSK
CTTNBP2
CUBN
CUL2
CUL3
CUL4B
CUL5
CUL7
CUL9
CX3CR1
CXCL5
CXCL6
CXCL12
CXCR1
CXCR2
CXCR4
CYB5A
CYB5R3
CYB5R4
CYBA
CYBB
CYBRD1
CYLD
CYP1A1
CYP1A2
CYP1B1
CYP2A6
CYP2A7
CYP2A13
CYP2B6

CYP2C8
CYP2C9
CYP2C18
CYP2C19
CYP2D6
CYP2E1
CYP2F1
CYP2J2
CYP2R1
CYP2U1
CYP3A4
CYP3A5
CYP3A7
CYP4A11
CYP4A22
CYP4F2
CYP4F3
CYP4F8
CYP4F11
CYP4F12
CYP4F22
CYP4V2
CYP7A1
CYP7B1
CYP11A1
CYP11B1
CYP11B2
CYP17A1
CYP19A1
CYP21A1P
CYP21A2
CYP24A1
CYP26B1
CYP26C1
CYP27A1
CYP27B1
CYS1
D2HGDH
DAD1
DAG1
DAO
DARS2
DAZ1
DAZ2

DAZ3
DAZ4
DAZL
DBH
DBT
DCAF17
DCBLD1
DCC
DCK
DCLRE1C
DCN
DCTD
DCTN1
DCX
DCXR
DDB2
DDC
DDHD1
DDHD2
DDI2
DDOST
DDR2
DDRGK1
DDX3X
DDX3Y
DDX25
DDX58
DEAF1
DEF6
DEFB126
DES
DGCR2
DGKD
DGKE
DGUOK
DHCR7
DHCR24
DHDDS
DHFR
DHH
DHODH
DHRS4
DHRS4L1
DHRS4L2

DHTKD1
DHX37
DIABLO
DIAPH1
DIAPH2
DIAPH3
DICER1
DIP2A
DIP2B
DIP2C
DIS3L2
DISC1
DISP1
DKC1
DKK3
DLAT
DLC1
DLD
DLEC1
DLG2
DLG3
DLG4
DLGAP3
DLL1
DLL3
DLX3
DLX5
DLX6
DMC1
DMD
DMGDH
DMP1
DMPK
DMRT1
DMXL1
DNAAF1
DNAAF2
DNAAF3
DNAH5
DNAH9
DNAI1
DNAI2
DNAJB2
DNAJB6

DNAJC5
DNAJC6
DNAJC17
DNAJC19
DNAJC30
DNAL1
DNASE1
DNASE1L1
DNASE1L3
DNASE2
DNM1
DNM1L
DNM2
DNMT1
DNMT3A
DNMT3B
DNMT3L
DOC2A
DOCK3
DOCK4
DOCK6
DOCK7
DOCK8
DOCK9
DOK1
DOK2
DOK5
DOK7
DOLK
DOLPP1
DOT1L
DPAGT1
DPCD
DPM1
DPM2
DPM3
DPP6
DPY19L2
DPYD
DPYS
DRD1
DRD2
DRD3
DRD4

DRD5
DSC2
DSC3
DSCAM
DSG1
DSG2
DSG3
DSG4
DSP
DSPP
DST
DTNA
DTNBP1
DUOX1
DUOX2
DUOXA1
DUOXA2
DUS4L
DVL2
DYM
DYNAP
DYNC1H1
DYNC2H1
DYRK1A
DYSF
E2F1
E2F4
E2F5
EARS2
EBAG9
EBF4
EBP
ECE1
ECE2
ECI1
ECM1
ECM2
ECSIT
EDA
EDA2R
EDAR
EDARADD
EDN1
EDN3

EDNRA
EDNRB
EFCAB5
EFEMP1
EFEMP2
EFHC1
EFHC2
EFNA5
EFNB1
EFR3A
EFTUD2
EGF
EGFR
EGLN1
EGR2
EHBP1
EHD2
EHHADH
EHMT1
EIF2AK3
EIF2B1
EIF2B2
EIF2B3
EIF2B4
EIF2B5
EIF2D
EIF2S3
EIF4E
EIF4G1
EIF4H
ELAC2
ELANE
ELF4
ELK1
ELK3
ELMOD2
ELN
ELOVL4
ELP2
ELP4
EMC4
EMD
EME1
EMG1

EMX1
EMX2
EN2
ENAM
ENG
ENOSF1
ENOX1
ENPP1
ENTPD1
EOMES
EP300
EPAS1
EPB41
EPB41L1
EPB41L2
EPB42
EPC2
EPCAM
EPHA2
EPHA3
EPHA5
EPHA7
EPHB2
EPHB6
EPHX1
EPHX2
EPM2A
EPO
EPOR
EPX
ERAP1
ERAP2
ERBB2
ERBB3
ERBB4
ERCC1
ERCC2
ERCC3
ERCC4
ERCC5
ERCC6
ERCC8
ERI2
ERLIN2

ERMAP
ERRFI1
ESCO2
ESPN
ESR1
ESR2
ESRRB
ETFA
ETFB
ETFDH
ETHE1
ETV4
ETV6
EVC
EVC2
EVI5
EWSR1
EXO1
EXO5
EXOC4
EXOSC3
EXPH5
EXT1
EXT2
EXTL1
EXTL2
EXTL3
EYA1
EYA4
EYS
EZH2
F2
F2RL1
F5
F7
F8
F9
F10
F11
F12
F13A1
F13B
FA2H
FAAH

FAAH2
FABP1
FABP2
FADD
FAH
FAM8A1
FAM20A
FAM20C
FAM47A
FAM47B
FAM47C
FAM83H
FAM91A1
FAM104A
FAM120A
FAM120AOS
FAM126A
FAM161A
FAM161B
FAM205A
FAN1
FANCA
FANCB
FANCC
FANCD2
FANCE
FANCF
FANCG
FANCI
FANCL
FANCM
FARS2
FAS
FASLG
FASN
FASTKD2
FBLN1
FBLN5
FBN1
FBN2
FBP1
FBXL6
FBXO7
FBXO11

FBXW4
FBXW7
FBXW11
FCER1G
FCGR2A
FCGR2B
FCGR2C
FCN1
FCN2
FCN3
FDFT1
FECH
FEM1A
FEM1B
FERMT1
FERMT3
FEZF2
FFAR1
FFAR4
FGA
FGB
FGD1
FGD3
FGD4
FGF2
FGF3
FGF8
FGF9
FGF10
FGF14
FGF23
FGFR1
FGFR2
FGFR3
FGFR4
FGFRL1
FGG
FH
FHIT
FHL1
FHL2
FIG4
FIGLA
FIP1L1

FKBP1A
FKBP4
FKBP5
FKBP6
FKBP8
FKBP10
FKBP14
FKRP
FKTN
FLCN
FLG
FLI1
FLNA
FLNB
FLNC
FLOT1
FLT1
FLT3
FLT4
FLVCR1
FLVCR2
FMN1
FMN2
FMO1
FMO2
FMO3
FMO4
FMO5
FMOD
FMR1
FN1
FN3K
FNTB
FOLH1
FOLR1
FOLR3
FOXA1
FOXA2
FOXA3
FOXC1
FOXC2
FOXD1
FOXD2
FOXD3

FOXE1
FOXE3
FOXF1
FOXF2
FOXG1
FOXH1
FOXI1
FOXI2
FOXI3
FOXJ1
FOXK1
FOXL2
FOXM1
FOXN1
FOXO1
FOXP1
FOXP2
FOXP3
FOXRED1
FPGS
FPGT-TNNI3K
FPR1
FPR2
FPR3
FRAS1
FREM1
FREM2
FREM3
FRK
FRMD5
FRMD7
FRMPD4
FRY
FRZB
FSBP
FSCB
FSCN2
FSHB
FSHR
FST
FSTL1
FTCD
FTHL17
FTO

FTSJ1
FUCA1
FUS
FUT1
FUT2
FUT3
FUT5
FUT6
FUT7
FUT8
FUZ
FXN
FXYD2
FXYD6-FXYD2
FYCO1
FZD3
FZD4
FZD6
FZD9
G6PC
G6PC2
G6PC3
G6PD
GAA
GABARAPL1
GABBR1
GABRA1
GABRA2
GABRA6
GABRB3
GABRD
GABRG1
GABRG2
GABRR1
GABRR2
GAD1
GAD2
GADD45A
GAK
GAL3ST1
GAL3ST2
GAL3ST3
GAL3ST4
GALC

GALE
GALK1
GALNS
GALNT2
GALNT3
GALNT5
GALNT6
GALNT7
GALNT8
GALNT9
GALNT11
GALNT12
GALNT13
GALNT14
GALNTL6
GALT
GAMT
GAN
GAP43
GAS1
GATA1
GATA2
GATA3
GATA4
GATA5
GATA6
GATAD1
GATM
GBA
GBE1
GBGT1
GC
GCDH
GCGR
GCH1
GCK
GCKR
GCLC
GCLM
GCM2
GCNT1
GCNT2
GDAP1
GDF1

GDF3
GDF5
GDF6
GDF9
GDI1
GDNF
GFAP
GFER
GFI1
GFI1B
GFM1
GFPT1
GFRA1
GFRA2
GGCX
GGH
GH1
GH2
GHR
GHRH
GHRHR
GHRL
GHSR
GIGYF2
GIMAP8
GIP
GIPC3
GIPR
GIT1
GJA3
GJA8
GJB1
GJB2
GJB3
GJB4
GJB6
GJC2
GJC3
GK
GLA
GLB1
GLCCI1
GLDC
GLE1

GLI2
GLI3
GLIS2
GLIS3
GLMN
GLO1
GLP1R
GLRA1
GLRB
GLRX5
GLUL
GLYCTK
GM2A
GMDS
GMPS
GNAI2
GNAI3
GNAQ
GNAS
GNAT1
GNAT2
GNB1L
GNB3
GNE
GNG7
GNMT
GNPAT
GNPTAB
GNPTG
GNRH1
GNRHR
GNS
GOLGA3
GOLGA5
GOLGA6L9
GON4L
GOPC
GORAB
GOSR2
GOT1
GP1BA
GP1BB
GP6
GP9

GPAM
GPATCH8
GPBAR1
GPC3
GPC4
GPC6
GPD1
GPD1L
GPD2
GPHN
GPI
GPNMB
GPR1
GPR12
GPR37
GPR132
GPR139
GPR143
GPS1
GPSM2
GPT
GRB10
GREM1
GRHL1
GRHL2
GRHPR
GRIA1
GRIA2
GRIA3
GRIA4
GRID1
GRID2
GRIK1
GRIK2
GRIK4
GRIN1
GRIN2A
GRIN2B
GRIN3A
GRIP1
GRK1
GRK4
GRM1
GRM5

GRM6
GRM7
GRN
GRPR
GRXCR1
GSC
GSE1
GSN
GSPT1
GSPT2
GSR
GSS
GSTA1
GSTA2
GSTA3
GSTA5
GSTM1
GSTM3
GSTO1
GSTP1
GSTZ1
GTF2H5
GTF2IRD1
GTF2IRD2
GTF2IRD2B
GUCA1B
GUCY2C
GUCY2D
GUCY2F
GUSB
GYG1
GYG2
GYPA
GYPB
GYPC
GYPE
GYS1
GYS2
H6PD
HABP2
HACE1
HACL1
HADH
HADHA

HADHB
HAL
HAMP
HAND1
HAND2
HAPLN1
HARS2
HAVCR1
HAX1
HBB
HBD
HBE1
HBM
HCCS
HCFC1
HCN1
HCN2
HCN3
HCN4
HCRT
HCRTR2
HDAC4
HDAC6
HDAC8
HDAC9
HDC
HDLBP
HDX
HEPACAM
HEPH
HERC1
HES7
HESX1
HEXA
HEXB
HEY1
HEY2
HFE
HGD
HGF
HGSNAT
HIBCH
HIF1A
HIGD2A

HINT1
HIP1
HK1
HK2
HLA-A
HLA-B
HLA-C
HLA-DPA1
HLA-DPB1
HLA-DQA1
HLA-DQA2
HLA-DQB1
HLA-DQB2
HLA-DRB1
HLA-DRB5
HLA-F
HLA-G
HLCS
HLX
HMBS
HMCN1
HMGA2
HMGCL
HMGCR
HMGCS2
HMMR
HMOX1
HMX1
HMX2
HMX3
HNF1A
HNF4A
HNMT
HNRNPH1
HNRNPH3
HNRNPU
HOGA1
HOXA1
HOXA2
HOXA3
HOXA4
HOXA10
HOXA11
HOXA13

HOXB1
HOXB6
HOXB7
HOXB8
HOXB13
HOXC13
HOXD4
HOXD10
HOXD13
HP
HPCAL4
HPD
HPGD
HPR
HPRT1
HPS1
HPS3
HPS4
HPS5
HPS6
HPSE2
HR
HRAS
HRG
HSD3B1
HSD3B2
HSD3B7
HSD11B1
HSD11B2
HSD17B3
HSD17B4
HSD17B10
HSF1
HSF4
HSP90AA1
HSPA9
HSPB1
HSPB3
HSPB6
HSPB7
HSPB8
HSPG2
HTN1
HTN3

HTR1A
HTR1B
HTR2A
HTR2B
HTR2C
HTR3A
HTR3B
HTR3C
HTR3D
HTR3E
HTR7
HTRA1
HTRA2
HTT
HUWE1
HYAL1
HYDIN
HYLS1
IAPP
ICAM1
ICAM4
ICOS
ID3
ID4
IDH1
IDH2
IDH3B
IDO1
IDS
IDUA
IER3IP1
IFIH1
IFITM1
IFITM2
IFITM3
IFITM5
IFNAR1
IFNG
IFNGR1
IFNGR2
IFRD1
IFT43
IFT80
IFT88

IFT122
IFT140
IFT172
IGBP1
IGF1
IGF1R
IGF2
IGF2BP2
IGF2R
IGFALS
IGFBP7
IGHMBP2
IGLL1
IGSF1
IHH
IKBIP
IKBKB
IKZF1
IKZF3
IL1B
IL1R1
IL1RAPL1
IL1RN
IL2RA
IL2RG
IL3
IL4
IL4I1
IL4R
IL6
IL6R
IL6ST
IL7R
IL9R
IL10
IL10RA
IL10RB
IL11
IL11RA
IL12B
IL12RB1
IL12RB2
IL13
IL17F

IL17RA
IL18RAP
IL20RB
IL21R
IL23R
IL31RA
IL36RN
ILDR1
ILK
IMMP2L
IMPDH1
IMPDH2
IMPG2
INF2
ING1
INHA
INMT
INPP5B
INPP5D
INPP5E
INS
INSL3
INSL6
INSR
INSRR
INVS
IPCEF1
IQCB1
IQCG
IQGAP2
IQGAP3
IQSEC2
IRAK3
IRAK4
IRF1
IRF4
IRF5
IRF6
IRF8
IRGM
IRS1
IRS2
IRS4
IRX1

IRX2
IRX3
IRX4
IRX5
IRX6
ISCU
ISL1
ITCH
ITGA1
ITGA2
ITGA2B
ITGA3
ITGA4
ITGA6
ITGA7
ITGA9
ITGAM
ITGB1
ITGB2
ITGB3
ITGB4
ITGB6
ITIH4
ITIH6
ITK
ITM2B
ITPA
ITPKC
ITPR1
ITSN2
IVD
IYD
JAG1
JAG2
JAK2
JAK3
JAM3
JMJD1C
JPH2
JPH3
JRK
JUN
JUNB
JUP

KALRN
KANK1
KAT2A
KAT6A
KAT6B
KATNAL2
KBTBD13
KCNA1
KCNA3
KCNA4
KCNA5
KCNA6
KCNAB1
KCNAB2
KCNC2
KCNC3
KCNC4
KCND2
KCND3
KCNE1
KCNE2
KCNE3
KCNE4
KCNH2
KCNH3
KCNH6
KCNIP1
KCNIP4
KCNJ1
KCNJ2
KCNJ4
KCNJ5
KCNJ6
KCNJ8
KCNJ10
KCNJ11
KCNJ12
KCNJ13
KCNJ18
KCNK3
KCNK18
KCNMA1
KCNMB1
KCNMB4

KCNN2
KCNN3
KCNQ1
KCNQ2
KCNQ3
KCNQ4
KCNQ5
KCNS1
KCNT1
KCNV1
KCNV2
KCTD7
KCTD13
KDM3A
KDM5A
KDM5C
KDM5D
KDM6A
KDM6B
KDR
KEL
KERA
KHDC3L
KHK
KIAA0100
KIAA0232
KIAA0319
KIF1A
KIF1B
KIF3C
KIF5A
KIF7
KIF11
KIF17
KIF21A
KIF22
KIF27
KIR2DL1
KIR2DL3
KIR2DL4
KIR2DS4
KIR3DL1
KIR3DL2
KIR3DL3

KIRREL3
KISS1
KISS1R
KIT
KITLG
KL
KLC1
KLF1
KLF6
KLF8
KLF10
KLF11
KLHDC8B
KLHL3
KLHL7
KLHL9
KLHL10
KLHL13
KLK1
KLK4
KLK12
KLKB1
KMT2D
KNG1
KPNA1
KRAS
KRT1
KRT2
KRT3
KRT4
KRT5
KRT6A
KRT6B
KRT6C
KRT8
KRT9
KRT10
KRT12
KRT13
KRT14
KRT16
KRT17
KRT18
KRT37

KRT38
KRT71
KRT72
KRT73
KRT74
KRT75
KRT76
KRT81
KRT82
KRT83
KRT85
KRT86
KRTAP1-1
KRTAP1-3
KRTAP1-5
KRTCAP3
KYNU
L1CAM
L2HGDH
L3MBTL1
LAMA1
LAMA2
LAMA3
LAMA4
LAMB1
LAMB2
LAMB3
LAMB4
LAMC2
LAMC3
LAMP2
LAMTOR2
LAS1L
LBR
LCA5
LCAT
LCE3B
LCE3D
LCE3E
LCE5A
LCK
LCN10
LCT
LDB1

LDB3
LDHA
LDHB
LDLR
LDLRAD2
LDLRAP1
LECT2
LEF1
LEFTY1
LEFTY2
LEMD3
LEP
LEPR
LETM1
LFNG
LGALS2
LGALS13
LGALS16
LGI1
LGI4
LGR5
LHB
LHCGR
LHFPL5
LHX3
LHX4
LHX5
LHX8
LIAS
LIF
LIFR
LIG1
LIG3
LIG4
LILRA1
LILRA2
LILRA3
LILRA4
LILRA5
LILRA6
LILRB1
LILRB2
LILRB3
LILRB5

LIM2
LIMK1
LINC00470
LINC00523
LINC01252
LIPA
LIPC
LIPG
LIPH
LIPI
LIPN
LITAF
LLGL1
LMAN1
LMBR1
LMBRD1
LMF1
LMNA
LMNB1
LMNB2
LMO2
LMO4
LMTK3
LMX1B
LOX
LOXHD1
LOXL1
LOXL3
LPA
LPAR6
LPIN1
LPIN2
LPIN3
LPL
LPP
LRAT
LRBA
LRCH2
LRFN5
LRIT3
LRP1
LRP1B
LRP2
LRP4

LRP5
LRP5L
LRP6
LRP8
LRPPRC
LRRC4
LRRC6
LRRC8A
LRRFIP2
LRRIQ1
LRRK2
LRRTM1
LRSAM1
LRTOMT
LTBP1
LTBP2
LTBP3
LTBP4
LTBR
LTC4S
LTN1
LUM
LY9
LY96
LYN
LYST
LYZ
LZTFL1
LZTS1
MACROD2
MAD1L1
MADD
MAEA
MAF
MAFA
MAFB
MAGEE2
MAGEL2
MAGI2
MAGT1
MAK
MAK16
MALT1
MAML2

MAMLD1
MAN1A2
MAN1B1
MAN2A1
MAN2B1
MANBA
MAOA
MAOB
MAP2
MAP2K1
MAP2K2
MAP2K3
MAP2K4
MAP2K7
MAP3K1
MAP3K8
MAP3K14
MAP3K15
MAP6
MAP7D1
MAPK1
MAPK8IP1
MAPK10
MAPT
MARS2
MARVELD2
MASP1
MASP2
MASTL
MAT1A
MATN3
MAX
MBD1
MBD3
MBD4
MBD5
MBL2
MBNL1
MBOAT7
MBTPS2
MC1R
MC2R
MC3R
MC4R

MCC
MCCC1
MCCC2
MCEE
MCF2L2
MCFD2
MCHR1
MCM4
MCM5
MCM6
MCM7
MCM9
MCOLN1
MCPH1
MDGA2
MDK
MDM2
MDN1
ME2
MECOM
MECP2
MED12
MED12L
MED13L
MED17
MED23
MED25
MEF2A
MEF2C
MEFV
MEGF8
MEGF10
MEGF11
MEIS1
MEN1
MEPE
MERTK
MESP1
MESP2
MEST
MET
METTL8
METTL21A
MFAP4

MFF
MFGE8
MFN2
MFRP
MFSD8
MGAT1
MGAT2
MGAT3
MGAT4A
MGAT4B
MGAT4C
MGAT5
MGAT5B
MGLL
MGMT
MGP
MGST2
MGST3
MICA
MICAL1
MICB
MID1
MIF
MINPP1
MIP
MIPOL1
MIR96
MIR182
MIR183
MITF
MKKS
MKS1
MLC1
MLH1
MLH3
MLLT3
MLLT10
MLLT11
MLPH
MLX
MLXIPL
MLYCD
MMAA
MMAB

MMACHC
MMADHC
MME
MMEL1
MMP1
MMP2
MMP3
MMP8
MMP9
MMP10
MMP13
MMP14
MMP20
MN1
MNX1
MOCOS
MOCS1
MOCS2
MOG
MOGS
MOK
MPC1
MPDU1
MPDZ
MPG
MPHOSPH8
MPI
MPL
MPLKIP
MPO
MPP6
MPST
MPV17
MPV17L
MPZ
MR1
MRAP
MREG
MRPL3
MRPL43
MRPL48
MRPS12
MRPS16
MRPS22

MRRF
MS4A1
MS4A2
MS4A12
MSH2
MSH3
MSH6
MSMO1
MSR1
MSRA
MSRB3
MST1
MST1R
MSTN
MSX1
MSX2
MTA1
MTA2
MTAP
MTFMT
MTHFD1
MTHFR
MTHFS
MTM1
MTMR2
MTMR9
MTMR14
MTNR1A
MTNR1B
MTO1
MTPAP
MTR
MTRR
MTSS1
MTTP
MUC5B
MUC7
MUC15
MUS81
MUSK
MUTYH
MVK
MXI1
MYB

MYBPC1
MYBPC3
MYC
MYCN
MYD88
MYEF2
MYF5
MYF6
MYH1
MYH2
MYH3
MYH4
MYH6
MYH7
MYH8
MYH9
MYH11
MYH13
MYH14
MYL1
MYL2
MYL3
MYLK
MYLK2
MYO1A
MYO1C
MYO1D
MYO1E
MYO1F
MYO3A
MYO5A
MYO5B
MYO5C
MYO6
MYO7A
MYO7B
MYO9B
MYO15A
MYOC
MYOCD
MYOD1
MYOM1
MYOT
MYOZ2

MYPN
MYT1
MYT1L
NAA10
NAALADL2
NAGA
NAGLU
NAGPA
NAGS
NAIP
NAPRT
NARS2
NAT1
NAT2
NAT8L
NAV2
NBAS
NBEA
NBEAL2
NBN
NBPF1
NCALD
NCAPD2
NCAPH2
NCF2
NCF4
NCKAP1
NCOA1
NCR3
NCS1
NCSTN
NDE1
NDN
NDOR1
NDP
NDRG1
NDST1
NDST2
NDST3
NDST4
NDUFA1
NDUFA2
NDUFA4
NDUFA6

NDUFA8
NDUFA9
NDUFA10
NDUFA11
NDUFA12
NDUFA13
NDUFAF1
NDUFAF3
NDUFAF4
NDUFAF5
NDUFAF6
NDUFAF7
NDUFB1
NDUFB3
NDUFB9
NDUFS1
NDUFS2
NDUFS3
NDUFS4
NDUFS5
NDUFS6
NDUFS7
NDUFS8
NDUFV1
NDUFV2
NDUFV3
NEB
NEBL
NEDD4
NEDD4L
NEFH
NEFM
NEGR1
NEIL1
NEIL2
NEK1
NEK8
NEU2
NEUROD1
NEUROG3
NEXN
NF1
NF2
NFATC2

NFATC3
NFATC4
NFE2L1
NFIA
NFIB
NFIC
NFIX
NFKB2
NFKBIA
NFKBIZ
NFU1
NGF
NGLY1
NHEJ1
NHLRC1
NHS
NICN1
NIN
NINJ1
NIP7
NIPA1
NIPAL4
NIPBL
NIPSNAP1
NIPSNAP3A
NIPSNAP3B
NKAIN2
NKX2-1
NKX2-4
NKX2-5
NKX2-6
NKX3-1
NKX3-2
NLGN1
NLGN2
NLGN3
NLGN4X
NLGN4Y
NLRP1
NLRP2
NLRP3
NLRP7
NLRP12
NLRP14

NLRX1
NME1
NME1-NME2
NME5
NME7
NME8
NMNAT1
NMT2
NMU
NNT
NOBOX
NOD1
NOD2
NODAL
NOG
NOL3
NOP10
NOP56
NOS1
NOS1AP
NOS2
NOS3
NOTCH1
NOTCH2
NOTCH3
NOX3
NPAS2
NPAS3
NPAT
NPC1
NPC1L1
NPC2
NPFFR2
NPHP1
NPHP3
NPHP4
NPHS2
NPL
NPM1
NPPA
NPPC
NPR2
NPSR1
NPY

NPY1R
NPY2R
NQO1
NR1D1
NR1H2
NR1H3
NR1H4
NR1I2
NR1I3
NR2C2AP
NR2E1
NR2F1
NR2F2
NR3C1
NR3C2
NR4A1
NR4A2
NR4A3
NR5A1
NRAS
NR0B1
NR0B2
NRCAM
NRG1
NRL
NRP2
NRTN
NRXN1
NRXN2
NRXN3
NSD1
NSDHL
NSMF
NSUN2
NSUN7
NT5C3A
NT5DC1
NT5E
NTF3
NTF4
NTHL1
NTNG1
NTRK1
NTRK2

NTRK3
NUAK1
NUBPL
NUDC
NUDT1
NUMA1
NUP62
NUP155
NUP214
NXF3
NXF5
NXNL1
NYX
OAS1
OAS2
OAT
OBSCN
OBSL1
OCA2
OCLN
OCRL
ODC1
OFD1
OGG1
OGT
OLFM2
OLIG2
OLR1
OPA1
OPA3
OPCML
OPHN1
OPLAH
OPN1SW
OPRL1
OPRM1
OPTC
OPTN
OR5AC2
OR5H1
OR5H6
OR5H14
OR5H15
OR13G1

OR52H1
OR52N4
ORC1
ORC4
ORC6
OSMR
OSTM1
OTC
OTOA
OTOF
OTOGL
OTX2
OVCH2
OXCT1
OXT
P2RX1
P2RX5
P2RX7
P2RY4
P2RY12
PABPC4L
PABPN1
PACRG
PACRGL
PACS1
PADI4
PAFAH1B1
PAFAH1B3
PAH
PAK3
PAK6
PALB2
PALLD
PAMR1
PANK2
PAPSS2
PARD3B
PARK7
PARL
PARP1
PASK
PAX1
PAX2
PAX3

PAX4
PAX5
PAX6
PAX7
PAX8
PAX9
PBX1
PC
PCBD1
PCBP3
PCCA
PCCB
PCDH11X
PCDH11Y
PCDH15
PCDH18
PCDH19
PCDHA1
PCDHA2
PCDHA3
PCDHA4
PCDHA5
PCDHA6
PCDHA7
PCDHA8
PCDHA9
PCDHA10
PCDHA11
PCDHA12
PCDHA13
PCDHAC1
PCDHAC2
PCDHB2
PCDHB3
PCDHB4
PCDHB5
PCDHB6
PCDHB7
PCDHB8
PCDHB9
PCDHB10
PCDHB11
PCDHB12
PCDHB13

PCDHB14
PCDHB15
PCK2
PCLO
PCM1
PCMT1
PCNT
PCOLCE
PCP4
PCSK1
PCSK5
PCSK9
PDCD1
PDCD10
PDE4B
PDE4D
PDE6A
PDE6B
PDE6C
PDE6G
PDE6H
PDGFB
PDGFRA
PDGFRB
PDGFRL
PDHA1
PDHB
PDHX
PDK1
PDLIM3
PDLIM4
PDP1
PDPK1
PDSS1
PDSS2
PDX1
PDYN
PDZD7
PECR
PEPD
PER1
PER2
PEX1
PEX2

PEX3
PEX5
PEX6
PEX7
PEX10
PEX11B
PEX12
PEX13
PEX14
PEX16
PEX19
PEX26
PFKM
PFN1
PGAM2
PGAM5
PGBD3
PGK1
PGK2
PGM1
PGR
PGRMC1
PHB
PHEX
PHF2
PHF3
PHF6
PHF8
PHF11
PHGDH
PHIP
PHKA1
PHKA2
PHKB
PHKG2
PHLPP2
PHOX2A
PHOX2B
PHYH
PI4KA
PICALM
PICK1
PIF1
PIGA

PIGL
PIGM
PIGN
PIGO
PIGR
PIGV
PIGZ
PIK3CA
PIK3CB
PIK3CD
PIK3R1
PIK3R2
PIK3R5
PIKFYVE
PIM1
PIN1
PINK1
PIP5K1C
PITPNA
PITPNM3
PITX1
PITX2
PITX3
PKD1
PKD2
PKHD1
PKLR
PKM
PKN3
PKP1
PKP2
PLA2G2A
PLA2G4A
PLA2G5
PLA2G6
PLA2G7
PLA2G10
PLAG1
PLAGL1
PLAU
PLCB1
PLCB4
PLCD1
PLCE1

PLCG2
PLCZ1
PLD2
PLEC
PLEKHG4
PLEKHG5
PLG
PLIN1
PLN
PLOD1
PLOD2
PLOD3
PLP1
PLP2
PLSCR3
PLTP
PML
PMM2
PMP22
PMS1
PMS2
PNKD
PNKP
PNLIP
PNMT
PNP
PNPLA1
PNPLA2
PNPLA6
PNPO
PNPT1
POC1A
POF1B
POFUT2
POGZ
POLB
POLD1
POLE2
POLG
POLG2
POLH
POLL
POLR1C
POLR1D

POLR2F
POLR3A
POLR3B
POLR3H
POLRMT
POMC
POMGNT1
POMGNT2
POMK
POMP
POMT1
POMT2
PON1
PON2
PON3
POP1
POR
PORCN
POSTN
POTEE
POTEF
POU1F1
POU3F4
POU4F2
POU4F3
POU5F1
POU5F1B
POU6F2
PPARA
PPARG
PPARGC1B
PPIB
PPM1B
PPM1D
PPM1E
PPM1G
PPM1K
PPOX
PPP1R1A
PPP1R3A
PPP1R3C
PPP1R12B
PPP1R17
PPP2R1B

PPP2R2A
PPP2R2B
PPP2R2C
PPP3CA
PPP3R1
PPT1
PQBP1
PRAF2
PRB1
PRB2
PRB3
PRB4
PRCC
PRCD
PRCP
PRDM5
PRDM7
PRDM9
PRDM16
PREPL
PRF1
PRG4
PRH1
PRICKLE1
PRICKLE2
PRKACA
PRKAG2
PRKAG3
PRKAR1A
PRKAR1B
PRKCA
PRKCG
PRKCH
PRKCSH
PRKDC
PRKG1
PRKG2
PRLH
PRLR
PRM1
PRM2
PRMT3
PRMT7
PRMT9

PRND
PRNP
PROC
PROCR
PRODH
PROK1
PROK2
PROKR1
PROKR2
PROM1
PROP1
PROS1
PROX1
PROZ
PRPF3
PRPF6
PRPF8
PRPF31
PRPH
PRPH2
PRPS1
PRPS1L1
PRR19
PRRT2
PRRX1
PRSS1
PRSS2
PRSS3
PRSS3P2
PRSS8
PRSS12
PRSS23
PRSS56
PRTG
PRX
PSAP
PSAT1
PSEN1
PSEN2
PSENEN
PSMA4
PSMA6
PSMB8
PSMC3IP

PSMD1
PSMD7
PSPH
PSPN
PSTPIP1
PSTPIP2
PTAFR
PTCD1
PTCH1
PTCH2
PTCHD1
PTCHD3
PTEN
PTF1A
PTGDR
PTGER2
PTGER4
PTGIR
PTGIS
PTGS1
PTGS2
PTH
PTH1R
PTHLH
PTK7
PTOV1
PTPN1
PTPN6
PTPN11
PTPN12
PTPN14
PTPN22
PTPRC
PTPRJ
PTPRK
PTPRN2
PTPRO
PTPRQ
PTPRT
PTS
PUS1
PUS3
PUS10
PXDN

PYCR1
PYGB
PYGL
PYGM
PYY
PZP
QDPR
QKI
RAB2A
RAB3GAP1
RAB3GAP2
RAB7A
RAB11FIP5
RAB18
RAB23
RAB25
RAB27A
RAB27B
RAB29
RAB39B
RABGGTA
RABL6
RAC1
RAC2
RAD21
RAD21L1
RAD50
RAD51
RAD51B
RAD51C
RAD51D
RAD52
RAD54B
RAD54L
RAF1
RAG1
RAG2
RAI1
RALGAPA1
RALGDS
RANBP2
RANGRF
RAP1GDS1
RAPSN

RARA
RARS2
RASA1
RASGEF1B
RASGRP1
RASGRP2
RAX
RAX2
RB1
RB1CC1
RBBP8
RBFOX1
RBFOX2
RBL1
RBL2
RBM10
RBM15
RBM20
RBM28
RBMXL2
RBMXL3
RBP4
RBPJ
RC3H1
RCBTB1
RCBTB2
RCHY1
RD3
RDH5
RDH8
RDH12
RDX
RECQL4
REEP1
REL
RELN
REN
REPS2
RET
RETN
RFC2
RFT1
RFX1
RFX2

RFX5
RFX6
RFX8
RFXANK
RFXAP
RGL1
RGMA
RGPD1
RGPD2
RGPD3
RGPD4
RGPD5
RGPD6
RGPD8
RGR
RGS2
RGS7
RGS9
RGS9BP
RHAG
RHBDF2
RHCE
RHD
RHO
RHOG
RHOH
RHPN2
RIC1
RIMS1
RIMS2
RIMS3
RIN2
RIOK2
RIPK4
RIT2
RLBP1
RMND1
RNASE4
RNASEH2A
RNASEH2B
RNASEH2C
RNASEL
RNASET2
RNF6

RNF113A
RNF135
RNF139
RNF168
RNF170
RNF212
RNF213
ROBO1
ROBO2
ROBO3
ROCK1
ROGDI
ROM1
ROR2
RORA
ROS1
RP1
RP1L1
RP2
RP9
RPA1
RPA4
RPE65
RPGR
RPGRIP1
RPGRIP1L
RPIA
RPL5
RPL6
RPL10
RPL11
RPL24
RPL35A
RPL36
RPL36A-HNRNPH2
RPL38
RPN2
RPS5
RPS6KA3
RPS6KL1
RPS15
RPS19
RPS24
RPS26

RPSAP52
RPTOR
RRAS2
RRH
RRM2B
RS1
RSC1A1
RSPH4A
RSPH9
RSPO1
RSPO4
RSRC1
RTN2
RTN4R
RTTN
RUFY2
RUNX2
RUNX3
RUVBL1
RXFP2
RXRA
RYK
RYR1
RYR2
SAA1
SAA2
SAA2-SAA4
SACS
SAG
SAGE1
SALL1
SALL4
SAMD9
SAMHD1
SAR1B
SARDH
SARM1
SARS2
SART3
SAT1
SATB2
SATL1
SBF2
SC5D

SCARB1
SCARB2
SCARF2
SCGB1A1
SCGB3A2
SCLT1
SCN1A
SCN1B
SCN2A
SCN2B
SCN3A
SCN3B
SCN4A
SCN4B
SCN5A
SCN7A
SCN8A
SCN9A
SCN10A
SCN11A
SCNN1A
SCNN1B
SCNN1D
SCNN1G
SCO1
SCP2
SCRIB
SCUBE2
SDC3
SDCCAG8
SDHA
SDHAF1
SDHAF2
SDHB
SDHC
SDHD
SEC23A
SEC23B
SEC63
SECISBP2
SELE
SELL
SELP
SELPLG

SEMA3A
SEMA3E
SEMA4A
SEMA4C
SEMA4G
SEMA6D
SEMA7A
SEMG1
SEMG2
SEPSECS
SERAC1
SERPINA1
SERPINA3
SERPINA6
SERPINA7
SERPINA10
SERPINB5
SERPINB6
SERPINB11
SERPINC1
SERPIND1
SERPINE1
SERPINF1
SERPINF2
SERPING1
SERPINH1
SERPINI1
SERPINI2
SESN2
SETBP1
SETD2
SETX
SEZ6
SEZ6L
SEZ6L2
SF3B1
SF3B4
SFRP1
SFTPA1
SFTPA2
SFTPB
SFTPC
SFTPD
SGCA

SGCB
SGCD
SGCE
SGCG
SGSH
SH2B1
SH2B3
SH2D1A
SH2D2A
SH3BP2
SH3GL1
SH3PXD2B
SH3TC2
SHANK2
SHANK3
SHARPIN
SHBG
SHH
SHMT1
SHOC2
SHOX
SHOX2
SHROOM3
SHROOM4
SI
SIAE
SIGLEC7
SIGLEC8
SIGLEC9
SIGLEC12
SIGLEC14
SIGMAR1
SIK3
SIL1
SIM1
SIM2
SIPA1
SIPA1L1
SIRT1
SIRT3
SIX1
SIX2
SIX3
SIX5

SIX6
SKI
SLA
SLA2
SLC1A1
SLC1A3
SLC1A5
SLC2A1
SLC2A2
SLC2A4
SLC2A9
SLC2A10
SLC3A1
SLC4A1
SLC4A3
SLC4A4
SLC4A7
SLC4A10
SLC4A11
SLC5A1
SLC5A2
SLC5A4
SLC5A5
SLC5A7
SLC5A11
SLC6A1
SLC6A2
SLC6A3
SLC6A4
SLC6A5
SLC6A6
SLC6A8
SLC6A9
SLC6A11
SLC6A12
SLC6A13
SLC6A18
SLC6A19
SLC6A20
SLC7A2
SLC7A5
SLC7A7
SLC7A9
SLC7A10

SLC7A11
SLC9A3
SLC9A3R1
SLC9A6
SLC9A9
SLC9B1
SLC10A1
SLC10A2
SLC11A1
SLC11A2
SLC12A1
SLC12A3
SLC12A4
SLC12A6
SLC13A2
SLC14A1
SLC15A1
SLC16A1
SLC16A2
SLC16A12
SLC17A3
SLC17A5
SLC17A8
SLC19A2
SLC19A3
SLC20A2
SLC22A1
SLC22A2
SLC22A3
SLC22A4
SLC22A5
SLC22A6
SLC22A9
SLC22A11
SLC22A12
SLC22A18
SLC22A18AS
SLC22A25
SLC24A1
SLC24A2
SLC24A5
SLC25A3
SLC25A4
SLC25A12

SLC25A13
SLC25A15
SLC25A19
SLC25A20
SLC25A22
SLC25A35
SLC25A38
SLC25A39
SLC26A1
SLC26A2
SLC26A3
SLC26A4
SLC26A5
SLC26A6
SLC26A9
SLC26A10
SLC27A1
SLC27A4
SLC27A5
SLC28A1
SLC28A2
SLC28A3
SLC29A1
SLC29A2
SLC29A3
SLC30A2
SLC30A5
SLC30A8
SLC30A10
SLC31A1
SLC33A1
SLC34A1
SLC34A2
SLC34A3
SLC35A1
SLC35C1
SLC35D1
SLC35F3
SLC35G2
SLC36A1
SLC36A2
SLC37A4
SLC39A4
SLC39A12

SLC39A13
SLC40A1
SLC41A1
SLC44A2
SLC45A2
SLC46A1
SLC47A1
SLC47A2
SLC52A1
SLC52A2
SLC52A3
SLCO1A2
SLCO1B1
SLCO1B3
SLCO1B7
SLCO1C1
SLCO2A1
SLCO2B1
SLCO5A1
SLFN5
SLIT1
SLIT3
SLITRK1
SLITRK5
SLITRK6
SLURP1
SLX4
SMAD1
SMAD2
SMAD3
SMAD4
SMAD5
SMAD6
SMAD7
SMAD9
SMAP1
SMARCA2
SMARCA4
SMARCAD1
SMARCAL1
SMARCB1
SMARCE1
SMC1A
SMC3

SMCHD1
SMG1
SMIM3
SMN1
SMN2
SMO
SMOC1
SMOC2
SMPD1
SMPD3
SMPX
SMS
SMUG1
SNAI2
SNAP25
SNAP29
SNAPC5
SNCA
SNCAIP
SNCB
SND1
SNIP1
SNRK
SNRNP200
SNRPN
SNTA1
SNTB1
SNTG2
SNURF
SNX1
SNX3
SNX10
SNX22
SOBP
SOCS3
SOD1
SOD2
SOD3
SOHLH1
SORCS1
SORL1
SORT1
SOS1
SOST

SOX1
SOX2
SOX3
SOX5
SOX6
SOX7
SOX8
SOX9
SOX10
SOX17
SOX18
SP5
SP7
SP8
SP9
SP110
SPAG8
SPAG17
SPANXN1
SPANXN5
SPAST
SPATA7
SPATA16
SPATA21
SPATA31C1
SPECC1
SPECC1L
SPEF2
SPG11
SPG21
SPI1
SPINK1
SPINK5
SPINT2
SPP1
SPR
SPRED1
SPRED2
SPRN
SPRY2
SPRY4
SPSB1
SPTA1
SPTAN1

SPTB
SPTBN1
SPTBN2
SPTBN5
SPTLC1
SPTLC2
SQSTM1
SRC
SRCAP
SRD5A3
SREBF1
SREBF2
SRGAP3
SRI
SRP72
SRPX
SRPX2
SRR
SRY
SSH1
SSPN
SSTR5
SSX1
SSX2
SSX3
SSX5
SSX7
ST3GAL1
ST3GAL2
ST3GAL3
ST3GAL4
ST3GAL5
ST3GAL6
ST6GAL1
ST6GAL2
ST6GALNAC1
ST6GALNAC2
ST6GALNAC3
ST6GALNAC4
ST6GALNAC5
ST8SIA1
ST8SIA2
ST8SIA3
ST8SIA4

ST8SIA5
ST8SIA6
ST14
ST20-MTHFS
STAR
STARD9
STAT1
STAT3
STAT4
STAT5A
STAT5B
STEAP3
STIL
STIM1
STK4
STK11
STK11IP
STK19
STK35
STK36
STK39
STON1-GTF2A1L
STOX1
STRA6
STRADA
STRC
STS
STT3A
STT3B
STX11
STX16
STXBP1
STXBP2
SUCLA2
SUCLG1
SUCO
SUFU
SUGCT
SULF1
SULT1A1
SULT1A2
SULT1C2
SULT2A1
SULT2B1

SULT4A1
SUMO1
SUMO4
SUOX
SUPT3H
SUPT16H
SURF1
SUZ12
SV2B
SYCE2
SYCP3
SYK
SYN1
SYN2
SYN3
SYNE1
SYNE2
SYNGR1
SYNM
SYNPO
SYNPR
SYP
SYT2
SYT14
SYT17
SYTL3
SYTL5
TAAR1
TAAR9
TAC3
TACO1
TACR3
TACSTD2
TAF1
TAF1C
TAF1L
TAF2
TAL1
TAL2
TALDO1
TAP2
TARDBP
TAS1R1
TAS1R3

TAS2R3
TAS2R16
TAS2R19
TAS2R20
TAS2R30
TAS2R31
TAS2R38
TAS2R43
TAS2R46
TAS2R50
TAT
TAZ
TBC1D4
TBC1D9
TBC1D23
TBC1D24
TBCD
TBCK
TBK1
TBL1X
TBL1XR1
TBL1Y
TBP
TBX1
TBX2
TBX3
TBX4
TBX5
TBX6
TBX10
TBX15
TBX19
TBX20
TBX21
TBX22
TBXA2R
TBXAS1
TCAP
TCF3
TCF4
TCF7L1
TCF7L2
TCF21
TCIRG1

TCN1
TCN2
TCOF1
TCP1
TCTE1
TCTN1
TCTN2
TCTN3
TDO2
TDP1
TDRD7
TEAD1
TEC
TECPR2
TECR
TECTA
TEK
TEKT2
TERT
TET1
TET2
TEX14
TF
TFAM
TFAP2A
TFAP2B
TFB1M
TFE3
TFF1
TFG
TFPI
TFR2
TFRC
TG
TGFB1
TGFB2
TGFB3
TGFBI
TGFBR1
TGFBR2
TGFBR3
TGIF1
TGM1
TGM2

TGM5
TGM6
TH
THAP1
THBD
THBS1
THBS2
THOC2
THPO
THRA
THRB
THSD7A
TICAM1
TIMM8A
TIMM44
TIMP3
TINAG
TINF2
TIRAP
TJP2
TK2
TLL1
TLR1
TLR2
TLR3
TLR4
TLR5
TLR6
TLR9
TLX1
TLX1NB
TLX2
TLX3
TM4SF19
TMC1
TMC6
TMC8
TMCO1
TMEM43
TMEM50B
TMEM67
TMEM70
TMEM99
TMEM114

TMEM126A
TMEM127
TMEM135
TMEM138
TMEM165
TMEM187
TMEM216
TMEM231
TMEM237
TMIE
TMLHE
TMPO
TMPRSS3
TMPRSS5
TMPRSS6
TMPRSS15
TMTC3
TNFRSF1A
TNFRSF1B
TNFRSF9
TNFRSF10A
TNFRSF10B
TNFRSF11A
TNFRSF11B
TNFRSF13B
TNFRSF13C
TNFSF4
TNFSF11
TNFSF14
TNKS
TNNC1
TNNI2
TNNI3
TNNI3K
TNNT1
TNNT2
TNNT3
TNP1
TNR
TNRC6B
TNS3
TNXB
TOE1
TOMM40L

TOP1
TOP2A
TOPORS
TOR1A
TP53
TP53BP1
TP53RK
TP63
TP73
TPCN2
TPH1
TPH2
TPK1
TPM1
TPM2
TPM3
TPMT
TPO
TPP1
TPR
TPRN
TPTE2
TRADD
TRAF3
TRAF3IP2
TRAF6
TRAK1
TRAPPC9
TRDN
TREH
TREM2
TRERF1
TREX1
TRHR
TRIB2
TRIB3
TRIM21
TRIM24
TRIM32
TRIM33
TRIM37
TRIO
TRIOBP
TRIP11

TRIP12
TRIP13
TRMU
TROAP
TRPA1
TRPC3
TRPC4
TRPC6
TRPM1
TRPM2
TRPM3
TRPM4
TRPM6
TRPM7
TRPS1
TRPV1
TRPV3
TRPV4
TRPV5
TRRAP
TSC1
TSC2
TSEN2
TSEN34
TSEN54
TSFM
TSG101
TSHB
TSHR
TSHZ1
TSLP
TSPAN7
TSPAN12
TSPAN17
TSPEAR
TSPO
TSPYL1
TSR1
TSSC4
TSSK4
TST
TTBK2
TTC7A
TTC8

TTC14
TTC19
TTC21B
TTC37
TTI2
TTLL1
TTN
TTPA
TTR
TUB
TUBA1A
TUBA3C
TUBA3D
TUBA3E
TUBA4A
TUBA8
TUBB
TUBB1
TUBB2A
TUBB2B
TUBB3
TUBB4A
TUBB4B
TUBB6
TUBGCP6
TUFM
TULP1
TUSC3
TWIST1
TWIST2
TXNRD2
TYK2
TYMP
TYMS
TYR
TYRO3
TYROBP
TYRP1
UACA
UBA1
UBA5
UBAC2
UBE2A
UBE2B

UBE2N
UBE3A
UBE3C
UBIAD1
UBQLN2
UBR1
UBR3
UBR7
UCHL1
UCN
UCP1
UCP2
UCP3
UGCG
UGGT1
UGGT2
UGT1A1
UGT1A3
UGT1A4
UGT1A5
UGT1A6
UGT1A7
UGT1A8
UGT1A9
UGT1A10
UGT2A2
UGT2A3
UGT2B4
UGT2B7
UGT2B10
UGT2B11
UGT2B15
UGT2B17
UGT2B28
UGT8
UIMC1
ULK4
UMOD
UMPS
UNC5C
UNC5CL
UNC13D
UNC80
UNC93A

UNC93B1
UNC119
UNG
UNKL
UPB1
UPF3B
UPK3A
UQCRB
UQCRFS1
UQCRQ
URB1
UROC1
UROD
UROS
USB1
USF1
USH1C
USH1G
USH2A
USP1
USP3
USP9X
USP9Y
USP15
USP26
USP46
UST
UTF1
UTP14C
UTRN
UTY
UVSSA
VAMP7
VANGL1
VANGL2
VAPB
VAX1
VCAM1
VCAN
VCL
VCP
VCX
VCX2
VCX3A

VCX3B
VCY
VCY1B
VDR
VEGFA
VEGFC
VHL
VIM
VIPAS39
VKORC1
VLDLR
VMA21
VNN1
VPS13A
VPS13B
VPS33B
VPS35
VPS37A
VPS54
VRK1
VRK2
VSIG4
VSX1
VSX2
VWF
WAS
WASF3
WDFY3
WDPCP
WDR4
WDR11
WDR13
WDR19
WDR35
WDR36
WDR45
WDR45B
WDR62
WDR72
WDR81
WFS1
WIPF1
WNK1
WNK4

WNT4
WNT5A
WNT7A
WNT10A
WNT10B
WRAP53
WRN
WT1
WWC1
WWOX
WWTR1
XBP1
XDH
XG
XIAP
XK
XKR6
XPA
XPC
XPNPEP2
XPNPEP3
XRCC1
XRCC2
XRCC3
XRCC4
XRCC5
XRCC6
XYLT1
XYLT2
YARS2
YBX2
YWHAE
YY1
YY1AP1
ZAN
ZAP70
ZBTB16
ZBTB18
ZBTB24
ZBTB25
ZBTB40
ZBTB41
ZC3H14
ZCCHC8

ZCCHC12
ZCCHC13
ZCCHC18
ZDHHC2
ZDHHC9
ZDHHC15
ZDHHC17
ZEB1
ZEB2
ZFAT
ZFHX3
ZFP36
ZFP36L1
ZFP36L2
ZFP69
ZFP90
ZFPM2
ZFYVE26
ZFYVE27
ZHX3
ZIC1
ZIC2
ZIC3
ZIC4
ZIC5
ZKSCAN5
ZMPSTE24
ZMYM3
ZNF41
ZNF75A
ZNF75D
ZNF80
ZNF81
ZNF175
ZNF213
ZNF276
ZNF335
ZNF419
ZNF420
ZNF423
ZNF433
ZNF469
ZNF480
ZNF500

ZNF513
ZNF526
ZNF592
ZNF644
ZNF674
ZNF711
ZNF750
ZNF773
ZPBP
ZPBP2
ACACA
ACBD6
ADRA2B
AGBL1
AHRR
CCDC39
CCL3
CCL5
CLN3
CSAG3
CYB561D2
CYFIP1
DACH1
DUX4L1
DUX4L2
DUX4L3
EPPK1
FBXO10
GAS2L2
GJA5
GOLGA6L10
GPIHBP1
GPR179
GSTT1
GTF2I
GUCA1A
HNF1B
IFNAR2
IKBKG
INS-IGF2
KRIT1
LHX1
MATR3
MRC1

MSMB
NBPF14
NCOA4
NEFL
NPHS1
NR2E3
NXF2
NXF2B
OMG
OR1B1
OR8K3
OR10X1
OR51G1
ORAI1
PCDHB16
PDE8B
PDE11A
RASSF5
RBP3
RGS5
RPS17
RUNX1
SCO2
SLC6A14
SLC25A53
SOGA3
SPANXN2
SPATA13
SPG7
SRD5A2
SSX2B
ST6GALNAC6
SUMF1
TAB2
TAF15
TBCE
TERC
TMEM249
TPTE
TUBB8
TUBGCP5
TXNIP
UBE2NL
UGT2A1

WNT3
ZNF365
HTT
AARS
FAM175A
GPR56
GPR98
KAL1
HMHA1
ARSE
ATP5J2-PTCD1
B3GALTL
B3GNT1
C16orf58
PTRF
MURC
GIF
CYR61
CTGF
WISP3
CD3EAP
CGB
ICK
ADCK3
C5orf42
IspD
ISPD
ST5
FAM69A
ATP5SL
DYX1C1
HEATR2
C9orf66
IKBKAP
DGCR14
FBXO18
WBSCR17
GARS
GARS
ADRBK2
DFNA5
HIST3H3
HIST2H3D
HIST2H3C

HIST2H3A
HARS
HDGFRP2
Hdgfrp2
HFE2
IMPAD1
KIAA1462
KARS
KCNE1L
KIAA1279
SETD8
SUV420H1
CASC5
LARGE
GYLTL1B
LINS
LOR
LOR
MESDC2
MUT
MRE11A
MKL1
GIF
PVRL1
PVRL4
KIAA2022
WHSC1
RNF219
C4orf26
LEPRE1
LEPREL1
C2orf71
TMEM8A
DFNB59
PARK2
FAM213B
FAM134B
C18orf8
TROVE2
KIAA0226
TMEM5
SEPN1
SHFM1
SEPT5

SEPT7
SEPT9
SEPT12
C19orf66
DIRC2
SPG20
TMEM173
FAM208A
T
TCTE3
TENC1
UFD1L
CIRH1A
KIAA1033
KIAA0196
DFNB31
YARS
LOC100144595
LOC100505549
LOC100271832
FAM58A
FAM58A
HIST1H2AE
HIST1H2AE
HIST1H3F
HIST1H3F
PALM2-AKAP2

Supplementary material 2:

Stress Related Genes Panel

AARS2, ABCA7, ABCB4, ABCB11, ABHD11, ACSL4, ADCY5, ADCYAP1R1, ADGRV1, ADH1C, ADNP, AFG3L2, AIMP1, AIP, ALAD, ALDH4A1, ALDH5A1, ALG14, ALKBH8, ALOX12, AMACR, ANG, ANXA11, AOX1, AP2S1, APC2, APOE, AR, ARID2, ARMC5, ARSA, ARSG, ARVCF, ASH1L, ATOX1, ATP1A3, ATP7B, ATP8B1, ATP13A2, ATRX, ATXN2, ATXN8OS, ATXN10, B3GALNT2, BAZ1B, BCL7B, BCL11B, BCR, BCS1L, BMPR1A, BNIP3, BPTF, BRD4, BUD23, C9orf72, C12orf4, C19orf12, CABP4, CACNA1A, CACNA1G, CACNA1H, CARS1, CASR, CAT, CBS, CC2D1A, CCNF, CCS, CDH2, CDH23, CDKN1A, CDKN1B, CDKN2B, CDKN2C, CDON, CEP78, CEP85L, CFAP410, CHCHD10, CHD7, CHD8, CHMP2B, CHRNA2, CHRNA4, CHRNB2, CIB2, CISD2, CLCN4, CLDN3, CLDN4, CLIP1, CLIP2, CLN5, CLN6, CLRN1, CLTRN, CNR1, COASY, COL7A1, COMT, COQ2, CPOX, CRADD, CRBN, CRH, CRKL, CSF1R, CTSF, STEEP1, CYGB, CYP27A1, DAO, DCPS, DCTN1, DDB1, DEAF1, DEPDC5, DGUOK, DHCR24, DISP1, DLAT, DLL1, DLST, DMPK, DNA2, DNAJC5, DNAJC6, DNAJC13, DNAJC30, DNMT1, DNMT3A, DPH1, DRD2, DRD3, DUOX1, DUOX2, DUSP1, DUSP6, DYRK1A, EDC3, EHMT1, EIF4G1, EIF4H, ELN, EMC10, EPAS1, EPCAM, EPHA4, EPHX2, EPM2A, EPX, ERBB4, ESPN, EZR, FA2H, FAN1, FBXO31, FGF8, FGF14, FGF17, FGFR1, FH, FIG4, FKBP5, FKBP6, FLII, FLT4, FMN2, FMO3, FMR1, FOXH1, FOXM1, FOXP1, FRRS1L, FTH1, FTSJ1, FUS, FUZ, GABRA1, GABRB3, GABRD, GABRG2, GALT, GAS1, GBA, GCH1, GCLC, GCLM, GDAP2, GIGYF2, GLA, GLE1, GLI2, GLT8D1, GLUD2, GM2A, GNA11, GNAS, GNRH1, GNRHR, GP1BB, GPR35, GPR101, GPX1, GPX2, GPX3, GPX4, GPX5, GPX6, GPX7, GRIK2, GRIN2A, GRM7, GRN, GSN, GSR, GSS, GTF2IRD1, GTF2IRD2, HARS1, HCN1, HDAC8, HIRA, HIVEP2, HLA-B, HLA-DQA1, HLA-DQB1, HLA-DRB1, HMBS, HMOX1, HNMT, HNRNPA1, HNRNPH2, HS6ST1, HSPA1A, HSPG2, HTR2A, HTRA2, HTT, IDUA, IGF1R, IQSEC1, IQSEC2, JMJD1C, JPH3, JRK, KANSL1, KCNJ2, KCNT1, KCTD17, KDM5B, KIF1B, KISS1, KISS1R, KMT2A, KMT2E, KPTN, KRAS, KRT1, LARP7, LAT2, LGI1, LIMK1, LINS1, LMAN2L, LMNB1, LPO, LRRK2, MAMLD1, MAN1B1, MAN2B1, MAPK1, MAPT, MAX, MBL2, MBOAT7, MDH2, MECP2, MED12L, MED23, MED25, MEN1, METTL23, METTL27, MIR17HG, MLH1, MLH3, MLXIPL, MMP1, MPO, MPV17, MSH2, MSH6, MSRA, MST1, MSTO1, MT-ATP8, MT-CO1, MT-CO2, MT-CO3, MT-ND1, MT-ND4, MT-ND5, MT-ND6, MT-TF, MT-TH, MT-TL1, MT-TL2, MT-TN, MT-TQ, MT-TS1, MT-TS2, MT-TW, MT3, MYO7A, NAGS, NCDN, NCF1, NCF2, NDP, NDST1, NEFH, NEK1, NEMF, NEXMIF, NF1, NFASC, NFIB, NFIX, NHLRC1, NIPBL, NKX2-1, NODAL, NONO, NOS2, NOTCH3, NOX4, NOX5, NPY, NQO1, NR1H4, NR3C1, NR4A2, NSD1, NSMF, NSUN2, NUDT1, OCRL, OPRL1, OPTN, OXR1, OXSR1, P4HA2, PAH, PAK3, PANK2, PARK7, PCDH15, PCDH19, PDCD1, PDE10A, PDGFB, PDGFRB, PDLIM1, PDZD7, PER2, PER3, PFN1, PGAP1, PHF21A, PHIP, PIGC, PIK3CA, PINK1, PLA2G6, PMS1, PMS2, PNKP, PODXL, POLG, POLG2, PON1, PON2, PON3, PPARGC1A, PPM1D, PPOX, PPP1CB, PPP2R2B, PPT1, PQBP1, PRDX2, PRDX5, PRDX6, PREX1, PRKACA, PRKAR1A, PRKAR1B, PRKCG, PRKN, PRNP, PROK2, PROKR2, PRPH, PRRT2, PRSS12, PSAP, PSEN1, PSMD12, PTCH1, PTPN22, PTS, PURA, RAD21, RAI1, RELN, RET, RFC2, RGS2, RIMS2, RLIM, RNF7, RNF125, ROS1, RPS6KA3, RPS20, RREB1, RRM2B, RSRC1, SARS1, SATB2, SCARA3, SCN1A, SCN1B, SCN2A, SCN9A, SDHA, SDHAF2, SDHB, SDHC, SDHD, SEC24C, SELENOP, SEMA4A, SEMA4D, SETD1A, SETD1B, SETD2, SETD5, SFTPD, SGCE, SHH, SIRT2, SIX3, SLC2A1, SLC2A3, SLC6A3, SLC6A4, SLC6A19, SLC7A6OS, SLC12A2, SLC18A2, SLC20A2, SLC25A4, SLC25A11, SLC35C1, SLC45A1, SMC1A, SMC3, SMPD1, SNCA, SNCAIP, SNRPN, SOD1, SOD2, SOD3, SOX5, SPART, SPAST, SPECC1L, SPRY4, SPTBN1, SQSTM1, SRCAP, SRPX2, SRXN1, ST3GAL3, STAG2, STK25, STUB1, STX1A, STX1B, STX16, STXBP1, SYNJ1, TAC3, TACR3, TAF1, TANC2, TARDBP, TBK1, TBL2, TBP, TBX1, TCF4, TCF20, TDGF1, TECR, TET3, TGFBR2, TGIF1, THOC2, TK2, TLK2, TMCO1, TMEM106B, TMEM127, TMEM270, TNIK, TOR1A, TP53, TPH1, TPO, TRAPPC9, TREM2, TREX1, TRHR, TSC1, TSC2, TSHB, TTC5, TTC19, TTN, TUSC3, TWNK, TXN, TXNRD1, TXNRD2, UBE4A, UBQLN2, UCHL1, UCP2, UFD1, UFSP2, UNC13A, UQCRC1, USH1C, USH1G, USH2A, USP8, VAPB, VCP, VHL, VPS13A, VPS13C, VPS35, VPS37D, WAC, WASHC4, WDR11, WFS1, WHRN, XK, XPR1, YWHAG, YY1, ZBTB20, ZC3H14, ZIC2, CLN3, MATR3, PDE11A, TBC1D7, GTF2I, TAF15, CCL5, GBA1, ENSG00000255292, ENSG00000258728, ENSG00000261832
